# Anion exchange on hydrous zirconium oxide materials: application for selective iodate removal†

**DOI:** 10.1039/d2ra06489h

**Published:** 2023-01-04

**Authors:** Valtteri Suorsa, Miho Otaki, Topi Suominen, Juhani Virkanen, Hanna Reijola, René Bes, Risto Koivula

**Affiliations:** a Radiochemistry Unit, Department of Chemistry, University of Helsinki A.I. Virtasen aukio 1 00014 Helsinki Finland valtteri.suorsa@helsinki.fi; b Department of Geosciences and Geography, University of Helsinki Gustaf Hällströmin katu 2 00014 Helsinki Finland; c Department of Physics, University of Helsinki PO Box 64 FI-00014 Helsinki Finland; d Helsinki Institute of Physics PO Box 64 FI-00014 Helsinki Finland

## Abstract

The radioactive ^129^I is a top-priority radionuclide due to its the long half-life (1.57 × 10^7^ years) and high mobility. Because of the planned and accidental releases to the environment, specific separation technologies are required to limit the potential radiation dose to human beings. Zirconium oxides are known for their adsorption capability and selectivity to oxyanions and here the applicability to selective IO_3_^−^ removal has been investigated regarding the uptake mechanism, regeneration and competition caused by other anions, like environmentally relevant SO_4_^2−^. Granular aggregates of hydrous zirconium oxides with and without Sb doping showed high potential for the selective IO_3_^−^ removal in the presence of competing anions, like the forementioned SO_4_^2−^ (apparent capacity between 0.1–0.4 meq g^−1^ depending on SO_4_^2−^ concentration). The main uptake mechanism was found to be outer-sphere complexation (ion-exchange) to the protonated hydroxyl groups of hydrous zirconium oxides, but also minor mechanisms were identified including inner-sphere complexation and reduction to I^−^. The materials were observed to be easily and successively regenerated using dilute acid. Hydrous zirconium oxides showed high potential for IO_3_^−^ removal from waste solutions regarding technical (high selectivity and apparent capacity) and ecological/economic (feasible regeneration) aspects.

## Abstract 2

Hydrous ZrO_2_ materials showed high potential for the selective adsorption of IO_3_^−^ in the presence of competing anions. The main adsorption mechanism was found to be ion-exchange and efficient regeneration was demonstrated with dilute acid.

## Graphical abstract

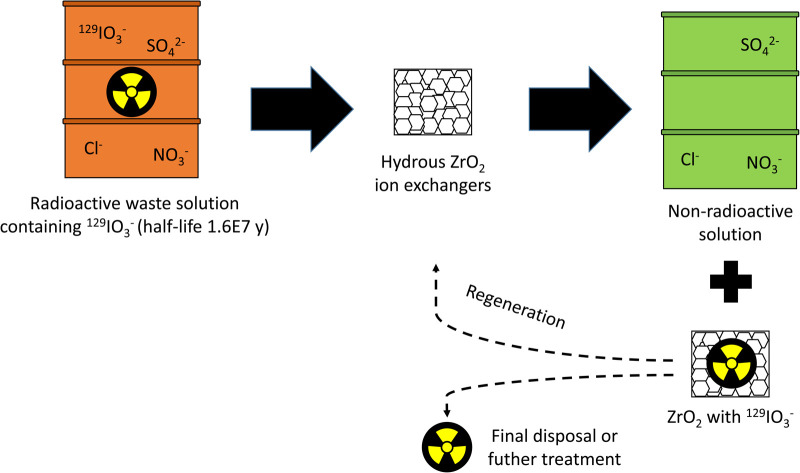

## 1. Introduction

Iodine is a vital element for human beings and other mammals for the proper functioning of the thyroid gland. Although the most abundant isotope of iodine is stable ^127^I, also radioactive isotopes are formed during uranium and plutonium fission. Like stable iodine, these radioactive isotopes also concentrate in the thyroid gland if inhaled or digested causing an elevated risk of thyroid cancer. The most relevant risks of the radioactive iodine isotopes can be divided into two categories: the long-term risk of ^129^I with an extremely long half-life (15.7 million years) and acute risk mainly from ^131^I with a short half-life (8 days). The former is important from an environmental perspective because of its long half-life and high mobility. Therefore, its risk must be assessed in the case of groundwater contamination and nuclear waste disposal. For example, ^129^I is regarded as one of the top priority radionuclides in the biosphere safety assessment of the final disposal of spent nuclear fuel in Finland.^1^^131^I is only important in the case of fresh fallout during, for instance, nuclear accidents like at the Chernobyl or Fukushima Daiichi nuclear power plants. The effects of acute iodine uptake and internal dose can be minimized by saturating the thyroid in advance with non-radioactive iodine for which iodine pills are intended.

Iodine has complex chemistry in the environment with its three main oxidation states −1, 0, +5. Iodate (IO_3_^−^) is the main species with its +5 oxidation state found in oxidizing environments, whereas iodide (I^−^) prevails with its −1 oxidation state in reducing conditions. For example, in anoxic waters, such as the Baltic sea, iodine exists mainly as I^−^,^2^ but in oxidizing waters such as oceans,^3^ IO_3_^−^ is the dominant species. Molecular iodine (I_2_), *i.e.* 0 oxidation state, is the major species only at low pH.^4^ In addition, iodine readily reacts with organic molecules forming a wide range of different organo-iodine compounds.^5,6^

Iodine removal is strongly dependent on its speciation and the immobilization of different iodine species has been comprehensively reviewed in literature.^4^ Silver-based materials have proved to be feasible for I^−^ decontamination due to the formation of AgI with extremely low solubility (*K*_sp_ = 8.5 × 10^−17^).^7–11^ The solubility of AgIO_3_ is however much higher (*K*_sp_ = 3.2 × 10^−8^) which makes silver-based materials inefficient in the removal of IO_3_^−^. In fact, the different affinity of I^−^/IO_3_^−^ to Ag^+^ can be utilized in analytical separations of these iodine species.^12^ Other removal techniques for I^−^ include for example ion exchange resins,^13^ activated carbons,^14^ organoclays^14–16^ and hydrotalcites.^17^ For the removal of IO_3_^−^, a wide range of different adsorbent materials have been studied including zero valent iron,^18^ hydroxyapatites^19,20^ and hydrotalcites.^17,21,22^ Despite the intensive research, no selective, highly performing state-of-art iodate removal technique has yet been established.

ZrO_2_ is an amphoteric metal oxide known for its cation and anion exchange capabilities depending on the solution pH.^23–25^ ZrO_2_ is a widely used material that can be doped with different cations like Y,^26,27^ Ce^28^ or Sb^29–31^ in order to enhance its mechanical, electronic or chemical properties such as the zeta potential of the material. ZrO_2_ have several crystal structures from which the monoclinic, tetragonal, and cubic phases can be formed in ambient pressures. The monoclinic structure is the most stable in low temperatures, but cubic and tetragonal structures can be stabilized either by the doping or by limiting the crystallite size, *i.e.* forming nanocrystalline materials. The properties of the crystal structures differ significantly regarding their physical and chemical properties like toughness or the number of anion and oxygen vacancies.^32^ Sb is known to stabilize the tetragonal form of ZrO_2_,^29^ which has been shown to be the active phase for the adsorption of different anions.^33–35^

Our previous work^31^ demonstrated the effectiveness of hydrous ZrO_2_ materials for selective iodate removal. However, the findings could not holistically reveal the mechanism behind the adsorption of IO_3_^−^ or other anions. Within this study, we have extensively studied the basic ion exchange properties of pure ZrO_2_ and antimony doped Zr(Sb)O_2_ with different anions and the basic adsorption experiments have been complemented with supportive XAS (X-ray Absorption Spectroscopy) measurements. The focus has been on the mechanism of IO_3_^−^ adsorption but also the competition of SO_4_^2−^, due to its relevance to environmental decontamination and strong affinity to adsorbent materials, has been investigated extensively. Different conditions like concentration and type of competing anions and the reversibility of the adsorption have been investigated to understand the mechanisms of IO_3_^−^ uptake on ZrO_2_ materials. The reversibility has not only significance in studying the mechanism of uptake but also regarding the regeneration of the materials, which is an important ecological and economic practical aspect regarding actual application of adsorbents in decontamination processes.

## 2. Materials and methods

### 2.1. Chemicals

The reagents used within the study were of analytical grade (Alfa Aesar, Sigma-Aldirch, Riedel de Häen) and were used as received. Deionized water (Type 1: 18.2 MΩ cm or Type 2: 15.0 MΩ cm at 25 °C, Milli-Q ^®^ Merck Millipore or) was used for the solutions in experiments. A radioactive Na^125^I (PerkinElmer) tracer was used as IO_3_^−^ probe after oxidation with NaOCl (final concentration ∼2 × 10^−4^ M). The speciation of ^125^I was confirmed with the method using silver-impregnated activated carbon (Silcarbon Aktivkohle GmbH, Germany).^12^ In brief, 10 mL of solution containing ^125^IO_3_^−^ was equilibrated for 24 h with 20 mg of the silver -impregnated activated carbon and the solid and the solution were separated. The radioactivity of the solution was measured and compared with the original solution. After successful oxidation, the radioactivity of the original and the separated solution were equal as IO_3_^−^ adsorption to the material is insignificant whereas I^−^ adsorption is extremely efficient. If the oxidation was not complete, more NaOCl was added, and the analysis step was repeated before use.

### 2.2. Synthesis of materials

The materials were synthesized and characterized as described earlier in literature.^31^ In brief, two different zirconium oxides were synthesized by the precipitation method. First, Sb doped Zr(Sb)O_2_ was synthesized by dissolving 45 g of ZrCl_4_ (Riedel de Häen) and 2 g of SbCl_3_ (Sigma-Aldrich) to 2 L of 3 M HCl under vigorous stirring using a mechanical stirrer. 1.2 L of 6 M NH_3_ was added slowly to the solution until pH reached 7.8 and white gel was precipitated. ZrO_2_ was synthesized similarly but here 100 g of zirconium basic carbonate (Alfa Aesar) was dissolved in 1 L of 6 M HNO_3_. Precipitates were let to stand in their mother solution overnight and clear supernatants were discarded. The precipitates were washed with deionized water until the conductivity of the supernatant was less than 4.0 mS cm^−1^. After the wash, the materials were dried in an oven at 70 °C for three days. The dried materials were ground and sieved to particle size 74–149 μm. The syntheses yielded large particles of amorphous zirconia.^31^

### 2.3. Aqueous sample analysis

#### 2.3.1 Iodine analyses

In most part of the experiments, radioactive ^125^IO_3_^−^ was used as an iodate probe. The concentration of IO_3_^−^ was adjusted by dissolving corresponding mass of non-radioactive K^127^IO_3_ to deionized water. After the desired solution was ready, it was spiked with ^125^IO_3_^−^ tracer solution (see Section 2.1. for the preparation and quality control procedure). The radioactivity of ^125^IO_3_^−^ in the experiments was 10–25 Bq mL^−1^ corresponding to the concentration of about 10^−13^ mol L^−1^. The routine radioactivity measurements were performed by measuring 5 mL of solution in 20 mL polyethylene scintillation vials with Wallac 1480 Wizard 3′′ automated NaI-scintillation *γ*-detector. On a few occasions, energy dispersive measurements were conducted with Canberra XtRa GX8021 germanium detector with a thin entrance window. A supportive experiment was performed with the non-radioactive iodine where analysis was done with an HPLC-ICP-MS (see ESI† or ref. 12 and 31 for more detailed description of the system).

#### 2.3.2 Other anion analyses

The other anions (Cl^−^, NO_3_^−^, and SO_4_^2−^) were measured with ion chromatography (IC) using Metrohm ECO IC instrument with Metrosep A Supp 5150/4.0 anion column connected to 858 Professional Sample Processor and using a mixture of 3.2 mM Na_2_CO_3_ and 1 mM NaHCO_3_ as an eluent. The anions were identified based on their retention times and the concentrations were calculated from the chromatogram peak areas compared with measured references.

### 2.4. Batch ion exchange experiments

In a typical batch experiment, 20 ± 1 mg of the ground and sieved material was weighed into a 20 mL polyethylene scintillation vial to which 10 mL of appropriate solution was pipetted. In some experiments larger masses and volumes were used but the batch factor, *i.e.* the solution to solid ratio, was kept the same at approximately 500 mL g^−1^. If needed, pH was adjusted with appropriate volumes of NaOH or HNO_3_. The samples were equilibrated in a rotary mixer for 24 h and the solids were separated from the liquid phase by a combination of centrifuging (2100 G, 10 min) and filtering with a 0.2 μm filter (PVDF LC. Arcodisc, Gellman Sciences). The equilibration time was chosen for practical reasons and to ensure that equilibrium was attained. Earlier studies^31^ have shown that IO_3_^−^ the adsorption on ZrO_2_ and Zr(Sb)O_2_ is in equilibrium before 1 hour and pH already after 5 minutes of contact. Finally, 5 mL of filtered solution was measured with a gamma counter as described in previous section (2.3.1 Iodine analyses) and the equilibrium pH measurement was conducted from the remaining filtered solution with an Orion™ 9103BNWP combination pH electrode (Thermo Scientific™). The uncertainty of pH measurements was estimated to be 0.1 units. All the experiments were conducted in normal laboratory air.

#### 2.4.1 Equilibrium pH experiment

The standard batch procedure described in the previous section was used to study equilibrium pH in deionized water, 5 mM and 50 mM solutions of NaNO_3_, NaCl, Na_2_SO_4_ and KIO_3_ with the initial pH of about 5.4–5.9 before the contact with Zr(Sb)O_2_ material. The samples containing the material and appropriate solutions were equilibrated in a rotary mixer for 24 h and solid and solution were separated, and pH of the solutions were measured.

#### 2.4.2 Successive batch equilibrations with NaNO_3_ solution

50 mg of ZrO_2_ and Zr(Sb)O_2_ were equilibrated three successive times with 50 mL of 10 mM NaNO3 (pH 5.6) solution in 50 mL polypropylene centrifuge tubes. The fourth equilibration was done with the same solution with ^125^IO_3_^−^ as a tracer to examine the uptake properties with NO_3_^−^ equilibrated material. The samples were done in duplicates. Between each equilibration, the samples were mixed in a rotary mixer for 24 h. After that the solid and solution were separated, pH of the solution was measured and solution was analysed with IC for the solution anion concentrations (Cl^−^, NO_3_^−^, SO_4_^2−^). Then, a fresh 50 mL batch of 10 mM NaNO_3_ was pipetted on the top of the material in the centrifuge tube. The procedure was repeated three times. After the third cycle, the material was equilibrated with ^125^IO_3_^−^ tracer (concentration about 10^−13^ mol L^−1^) without any stable ^127^IO_3_^−^ in 10 mM NaNO_3_. After 24 h in rotary mixer, the solid and solution were separated, pH of the solution was measured and ^125^IO_3_^−^ in the solution was measured with a gamma counter.

### 2.5. Column experiments

In a typical column experiment 0.2–0.5 g of the sieved (74–149 μm) material was weighed to a beaker and rinsed with deionized water. The water was decanted and discarded and remaining white solid was mixed with a small volume (∼5 mL) of fresh deionized water and transferred with a disposable pipette into a low-pressure borosilicate glass column with a diameter of 0.5 or 0.7 cm and a porous polymer bed support at the bottom (Econo-Column^®^. Bio-Rad Laboratories, Inc.). The feed solution was pumped through the column from the inlet with a flow velocity of about 20 bed volumes (BV) per hour and sample fractions were collected from the outlet. In columns with a smaller mass, a higher flow velocity of 40 BV per hour was used. From the collected fractions, pH and probe (mainly IO_3_^−^ but also NO_3_^−^, Cl^−^ and SO_4_^2−^ in some experiments) concentrations were measured with corresponding methods described earlier in Section 2.3. The feed solution pH and concentration of the elements of interest were monitored throughout the experiments.

#### 2.5.1 pH equilibration column experiment

Two columns were prepared for ZrO_2_ and one for Zr(Sb)O_2_ using 0.5 g of the material for each column. One of the ZrO_2_ columns was fed with deionized water with pH 5.6 and the rest were fed with 1 mM KIO_3_ solution with the same pH. The effluent was collected with a fraction collector using 120 min collection time before pH measurement.

#### 2.5.2 Column load and desorption experiments

Three different series of column adsorption experiments were performed within the study. In the first set of experiments, 0.2 g columns of ZrO_2_ and Zr(Sb)O_2_ were loaded with 10 mM SO_4_^2−^, Cl^−^ and NO_3_ solutions containing ^125^IO_3_^−^ tracer with 1 mM of ^127^IO_3_^−^ carrier using a flow rate of about 40 BV per hour to study IO_3_^−^ uptake in the presence of different anions. A second set of experiments were performed with varying SO_4_^2−^concentrations (0.1–10 mM) while maintaining previous IO_3_^−^ concentration, column mass and flow rate. The experiment was used to assess the effect of SO_4_^2−^concentration on IO_3_^−^ uptake. After the loading, the columns were rinsed with about 15 mL of deionized water before the desorption of IO_3_^−^ with 0.1 M NaOH. Finally, pH and the radioactivity of ^125^IO_3_^−^ of the samples were measured. In the third experiment, the desorption of synthesis derived anions from the materials during SO_4_^2−^ adsorption was studied by loading columns with 1 mM Na_2_SO_4_ and measuring the anion (Cl^−^, NO_3_^−^, SO_4_^2−^) concentration of the effluent.

#### 2.5.3 Column elution experiments

0.5 g columns of Zr(Sb)O_2_ and ZrO_2_ were first loaded with 10 mM IO_3_^−^ followed by consecutive elutions with different solutions. The experiments were repeated twice. In the first experiment the elution solutions were 100 mM NaNO_3_, Na_2_SO_4_ and NaOH and in the second experiment only 100 mM Na_2_SO_4_ and NaOH were used. Every elution step was continued until the desorption of IO_3_^−^ was negligible and after that the eluent was changed. The fractions were collected with a fraction collector and finally pH and the radioactivity of ^125^IO_3_^−^ of the samples were measured.

#### 2.5.4 Regeneration experiments in column

Two sets of regeneration experiments were performed within the study. In the first experiment, 0.2 g columns of Zr(Sb)O_2_ and ZrO_2_ were first loaded with a solution containing 10 mM Na_2_SO_4_ and 10 μM KIO_3_ traced with ^125^IO_3_^−^ followed by desorption with 0.1 M NaOH. After desorption, the columns were washed with deionized water until pH was 5.5, to remove the remaining NaOH solution and the columns were treated with solutions containing 1 M NaCl or 0.1 M of either NaOH or HCl until pH of the feed and the effluent were the same. After washing the columns with a few BV of deionized water, they were loaded again with the same 10 mM Na_2_SO_4_ and 10 μM KIO_3_ solution and uptake profiles were measured. In the second experiment, the regeneration experiment was performed with the same 10 mM Na_2_SO_4_ solution but with higher KIO_3_ concentration (1 mM). Only 0.1 M HCl was used as the regeneration solution and in total the material was regenerated three times and its IO_3_^−^ uptake performance was investigated.

### 2.6. Solid sample characterization

#### 2.6.1 X-ray absorption spectroscopy

The oxidation states and the local coordination environment of I, Zr and Sb were determined by X-ray Near-Edge Structure (XANES) and Extended X-ray Absorption Fine structure (EXAFS) at their K-edges respectively situated at 33.169 keV, 17.998 keV and 30.491 keV. The measurements were done before (Zr and Sb only) and after (Zr, Sb and I) IO_3_^−^ adsorption in order to see the possible changes in their speciation. The XAS measurements were performed at PETRA III P64 beamline, the Deutsches Elektronen-Synchrotron (DESY), Germany. The spectra were collected in transmission (all the reference materials and Zr measurements) or fluorescence (I and Sb measurements of the samples) mode depending on the concentration level of the probe element. The sample temperature was maintained below 10 K using an He cryostat to minimize the Debye–Waller factor (thermal effects) and to reduce any potential beam damage. Energy calibration was achieved using a Zr foil, a Sb foil and an I_2_ cellulose sample for Zr, Sb and I respectively. The spectra of Sb_2_O_3_, Sb_2_O_5,_ I_2_, KI, KIO_3_ were also collected as references. The collected data was normalized, analyzed and repeated scans were merged with Athena software^36^ and finally compared with the measured data of the reference materials. EXAFS data analyses were done with Artemis software package^36^ using first shell fitting for I (*R*-space 1.1–1.75 Å based on *k*-space 2.8–12.5 Å^−1^), Sb (*R*-space 1.25–2 Å based on *k*-space 3–8 Å^−1^) and Zr (*R*-space 1.1–2.0 Å based on *k*-space 3–12 Å^−1^). The small ranges in *R*-space were selected as the first shell fitting does not require a large range and as extending the range only resulted in the addition of noise. Also, the larger ranges increased *R*-factors of the fittings without significantly changing the structural parameters within the reasonable values. The *k*-space ranges were selected by taking the maximum available range without significant amount of noise and considering the fact that the signal of the first shell damped after about 10 Å^−1^.

#### 2.6.2 Specific surface area measurements

Samples were analyzed with nitrogen physisorption at 77 K. Specific surface areas were calculated from the adsorption branch using the Brunauer–Emmett–Teller (BET) method and the total pore volume and average pore size were calculated from the desorption branch using the Barrett–Joyner–Halenda (BJH) method. The measurements were performed in an external laboratory as a service according to the standard ISO 9277:2010 using nitrogen adsorption at the temperature of liquid.

#### 2.6.3 Thermogravimetric analysis

Thermogravimetric analysis (TGA) of the materials was performed with STA 449F3 Jupiter, Netzsch instrument connected to JAS-Agilent gas chromatography-mass spectrometer (7890B GC/MSD5977A). In the TGA experiments, about 25 mg of dried sample was weighed on Al_2_O_3_ 70 μL crucibles. The samples were heated to 1200 °C under constant He flow with the heating rate of 20 °C min^−1^.

## 3. Results and discussion

For clarity and readability, the results are divided into two parts: (A) basic anion exchange studies regarding ZrO_2_ and Zr(Sb)O_2_ interactions with anions in general and (B) the selective adsorption of IO_3_^−^ to the materials regarding the adsorption mechanism and the effect of competing anions.

### 3.1. Basic anion exchange mechanisms on ZrO_2_ materials

The basic ion exchange phenomenon on ZrO_2_ and Zr(Sb)O_2_ was studied with the series of batch and column experiments, where anion exchange equilibrium of ZrO_2_ and Zr(Sb)O_2_ with OH^−^, Cl^−^, NO_3_^−^, SO_4_^2−^ and IO_3_^−^ was investigated.

#### 3.1.1 Equilibrium pH experiment

The equilibrium pH of Zr(Sb)O_2_ with different salt solutions were studied in batch experiments to understand the fundamentals of equilibrium ion exchange reactions between the material and solution (Table 1). In deionized water, the contact with the material lowered the pH from 5.4 (deionized water in equilibrium with the atmospheric CO_2_) to 3.3. Similarly, in NaCl and NaNO_3_ solutions the pH dropped from ∼5.5 to 3.4 and 3.7 in 5 mM and 10 mM solutions, respectively. A completely different behaviour was observed with Na_2_SO_4_ and KIO_3_, where the drop was negligible in 5 mM concentration and the pH rose to 5.9 and 6.4 in 50 mM solution, respectively.

**Table 1:** The equilibrium pH of Zr(Sb)O_2_ with different solutions. The initial solution pH values before the contact were 5.6 ± 0.2

Solution	Eq. pH DI water	Eq. pH 5 mM	Eq. pH 50 mM
NaCl	3.3	3.4	3.7
NaNO_3_	3.3	3.4	3.7
Na_2_SO_4_	3.3	5.4	5.9
KIO_3_	3.3	5.2	6.4

Fundamentally, the pH drop can be due either an increase in the concentration of H_3_O^+^ or the decrease of the concentration of OH^−^ in the solution. Because pH behaviour was different between the anions (and not the cations) the most probable reason for the pH drop is anion exchange between the OH^−^ of the solution and the exchangeable anions in the materials originated from the synthesis. Evidently, the solutions containing SO_4_^2−^ and IO_3_^−^ ions prevented this drop of pH, probably because they are exchanged instead of OH^−^, and in higher concentrations the equilibrium pH even rose which indicates they are exchanged to OH^−^ of the material thus indicating higher selectivity. The higher 50 mM concentration of Cl^−^ and NO_3_^−^ resulted only in a slightly higher equilibrium pH (3.7) compared to the 5 mM concentration (pH 3.4) and this is likely because with higher concentration more Cl^−^/NO_3_^−^ is adsorbed instead of OH^−^ but the selectivity is much lower compared with SO_4_^2−^ and IO_3_^−^.

#### 3.1.2 Successive batch equilibrations with NaNO_3_ solution

**Fig. 1:**
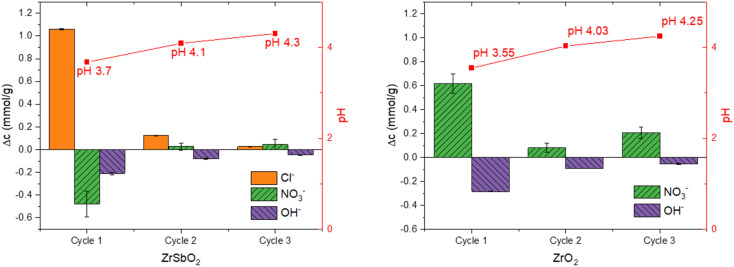
Anions adsorbed and desorbed during successive equilibrations of Zr(Sb)O_2_ and ZrO_2_ with 10 mM NaNO_3_ (pH 5.6). The negative values represent adsorption to the material and the positive values represent desorption to the solution phase. The standard deviations of pH measurements are now showing in figure as they were extremely small related to the measured value.

Zr(Sb)O_2_ and ZrO_2_ were equilibrated three successive times with NaNO_3_ solution (Fig. 1) to assess the balance between the adsorbed and desorbed anions. Zr(Sb)O_2_ released in total 1.21 ± 0.01 mmol g^−1^ of Cl^−^ (almost 90% already with the first equilibration) to the solution while 0.41 ± 0.13 mmol g^−1^ of NO_3_^−^ was adsorbed, whereas in ZrO_2_ 0.91 ± 0.17 mmol g^−1^ NO_3_^−^ was released to the solution. At the same time, pH was lowered from the original 5.6 to 3.6–4.3 depending on the fraction. Considering the pH change, the ratio of adsorbed (NO_3_^−^ and OH^−^) and desorbed (Cl^−^) anions was 1.0 ± 0.2 for Zr(Sb)O_2_ and similarly 1.3 ± 0.4 for ZrO_2_. Cl^−^ originates from the synthesis of Zr(Sb)O_2_ which was done in HCl (see Section 2.2. Synthesis of materials) and Cl^−^ seems to be released due the ion exchange between it and NO_3_^−^ and OH^−^ from the solution. ZrO_2_ instead, was synthesized in HNO_3_ which is the source of the released NO_3_^−^ ions. As the experiment was done in NaNO_3_ solution, only OH^−^ is exchanged with NO_3_^−^ in the material.

The ion exchange reaction is described by eqn (1) for Cl^−^ exchange (similarly with NO_3_^−^ in the case of ZrO_2_):

1 2H_2_O + Zr – OH_2_^+^Cl^−^ ⇌ H_3_O^+^ + Cl^−^ + Zr – OH_2_^+^OH^−^

In the exchange reaction, a neutral water molecule is exchanged to hydrochloric (or nitric) acid, which causes the decrease of pH.

The ion exchange between NO_3_^−^ and Cl^−^ is then described by eqn (2):

2 Na^+^ + NO_3_^−^ + Zr – OH_2_^+^Cl^−^ ⇌ Na^+^ + Cl^−^ + Zr – OH_2_^+^NO_3_^−^

After the successive batch equilibrations with NaNO_3_, both materials still showed high affinity to IO_3_^−^ in trace concentration in the same 10 mM NaNO_3_ solution: K_d_'s of IO_3_^−^ were 240 000 ± 3000 mL g^−1^ for ZrO_2_ (sorption-%: 99.58 ± 0.01) and 150 000 ± 30 000 mL g^−1^ for Zr(Sb)O_2_ (sorption-%: 99.30 ± 0.16).

#### 3.1.3 pH equilibration column experiment

**Fig. 2:**
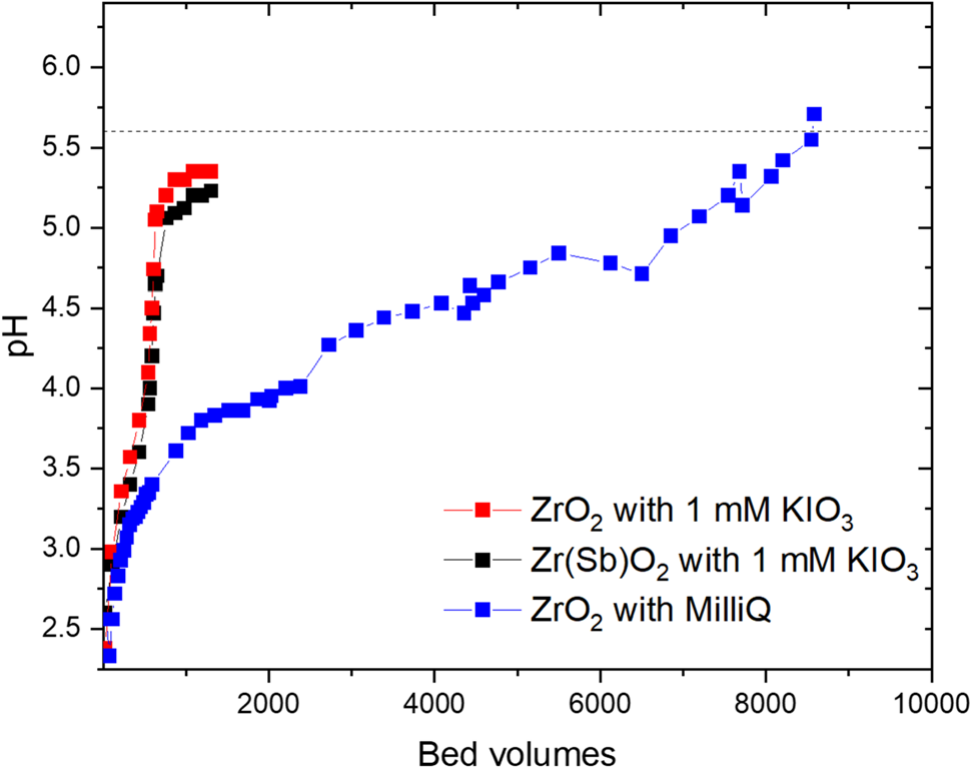
pH of eluate in ZrO_2_ with 1 mM KIO_3_ (red squares), Zr(Sb)O_2_ with 1 mM KIO_3_ (black squares) and ZrO_2_ with deionized water (blue squares). The pH of the feed was 5.6 and is shown as dashed horizontal line. The uncertainty of individual pH measurement was estimated to be 0.1 units.

The pH equilibration profiles of Zr(Sb)O_2_ and ZrO_2_ were studied in column experiments (Fig. 2) with 1 mM KIO_3_ solution and with deionized water (only for ZrO_2_) with pH 5.6 to investigate the attainment of pH equilibrium between the material and feed solution in different conditions. At the beginning of the experiment, the pH dropped to below 2.5 in all the experiments and rose steadily close to the pH of the feed. The plateau or equilibrium was reached after 1000 and 8000 BV's in KIO_3_ and deionized water, respectively.

The total OH^−^ uptake calculated from Δ[OH^−^] + Δ[H_3_O^+^] would equal to 0.83 ± 0.04 mmol g^−1^, which is well in line with the maximum uptake determined in the batch experiments (0.91 ± 0.17 mmol g^−1^) for other anions. These values agree also well with the apparent capacities published for hydrous zirconium oxides in literature.^23,37,38^ The decrease of pH is different in the presence of IO_3_^−^ as the latter seems to inhibit the pH lowering capability of zirconia materials. This indicates that OH^−^ and IO_3_^−^ compete for the same sites on the material surface.

#### 3.1.4 Column sorption/desorption experiment

The fundamental anion exchange properties were tested further with a column adsorption experiment, where the sorption of SO_4_^2−^ and simultaneous desorption of synthesis derived anions (OH^−^, NO_3_^−^, Cl^−^) were investigated for Zr(Sb)O_2_ and ZrO_2_.

**Fig. 3:**
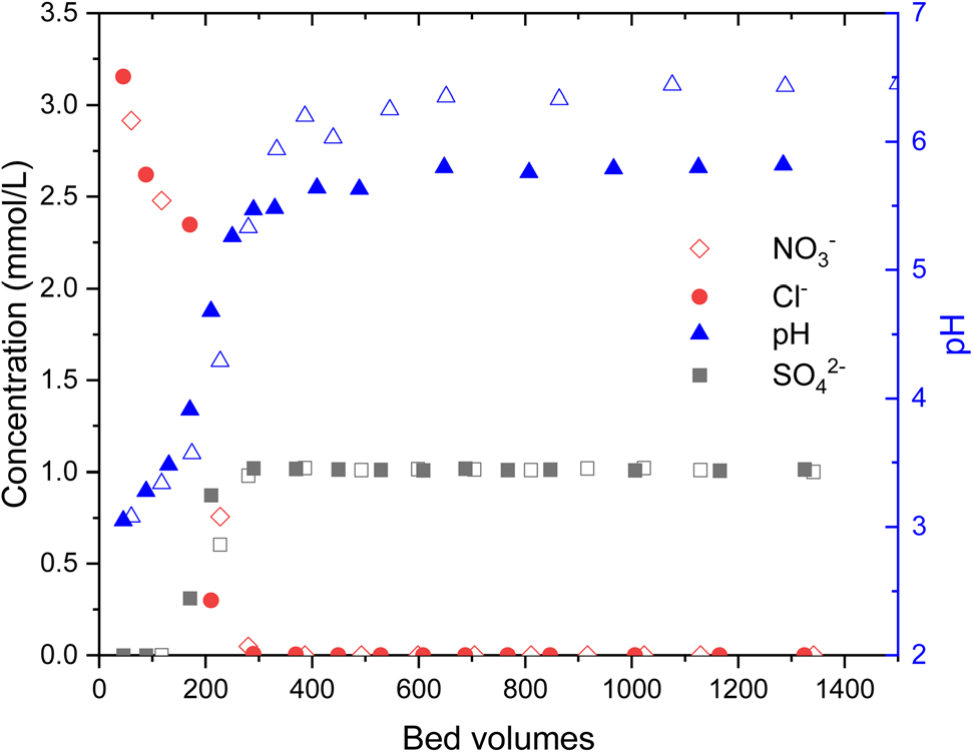
The effluent pH (blue symbols and right *y*-axis) and concentrations of SO_4_^2−^ (black symbols) and Cl^−^ (red symbols) for Zr(Sb)O_2_ (closed symbols) and SO_4_^2−^ and NO_3_^−^ for ZrO_2_ (open symbols). The uncertainties are not shown in the graph due to the clarity and were 5% (NO_3_^−^), 3% (Cl^−^) and 4.5% (SO_4_^2−^) of the reported concentration for individual samples.

During the loading of materials with of 1 mM Na_2_SO_4_, the release of synthesis derived anions from the materials were studied (Fig. 3). In total 0.49 ± 0.03 meq g^−1^ SO_4_^2−^ was exchanged to Cl^−^ (0.73 ± 0.02 mmol g^−1^) and OH^−^in the case of Zr(Sb)O_2_. During the first 300 BV's, pH steadily increased and stabilized to pH 5.6 after an instant drop to pH 3 at the beginning of the experiment. The ratio of adsorbed (SO_4_^2−^) and desorbed (Cl^−^ and OH^−^) equaled about 1.1. In total, 0.33 ± 0.01 meq g^−1^ of SO_4_^2−^ was exchanged to NO_3_^−^ (0.53 ± 0.02 mmol g^−1^) and OH^−^ in the case of ZrO_2_ which equals adsorption/desorption ratio of 1.25. Compared with Zr(Sb)O_2_, pH stabilized to about 6.5 that is slightly higher than the feed solution pH.

### 3.2. Application on selective IO_3_^−^ removal from waste solutions

The applicability of ZrO_2_ and Zr(Sb)O_2_ materials for selective IO_3_^−^ removal was studied with a series of column experiments with competing ions at different concentrations. In the first set of experiments, the competition of Cl^−^, NO_3_^−^ and SO_4_^2−^ with IO_3_^−^ were studied in respect to the total uptake and breakthrough (BT) profile of IO_3_^−^. In the second set, the same was done with SO_4_^2−^ with varying concentrations in the range of 0.1–10 mM. In the third set, the desorption of IO_3_^−^ from the loaded materials were studied with sequential elution with different solutions (100 mM NaNO_3_, Na_2_SO_4_ and NaOH). Finally, the regeneration of the materials was studied with different solutions (NaCl, NaOH and HCl) and then tested for four successive regeneration cycles.

#### 3.2.1 Column load and desorption experiments

**Fig. 4:**
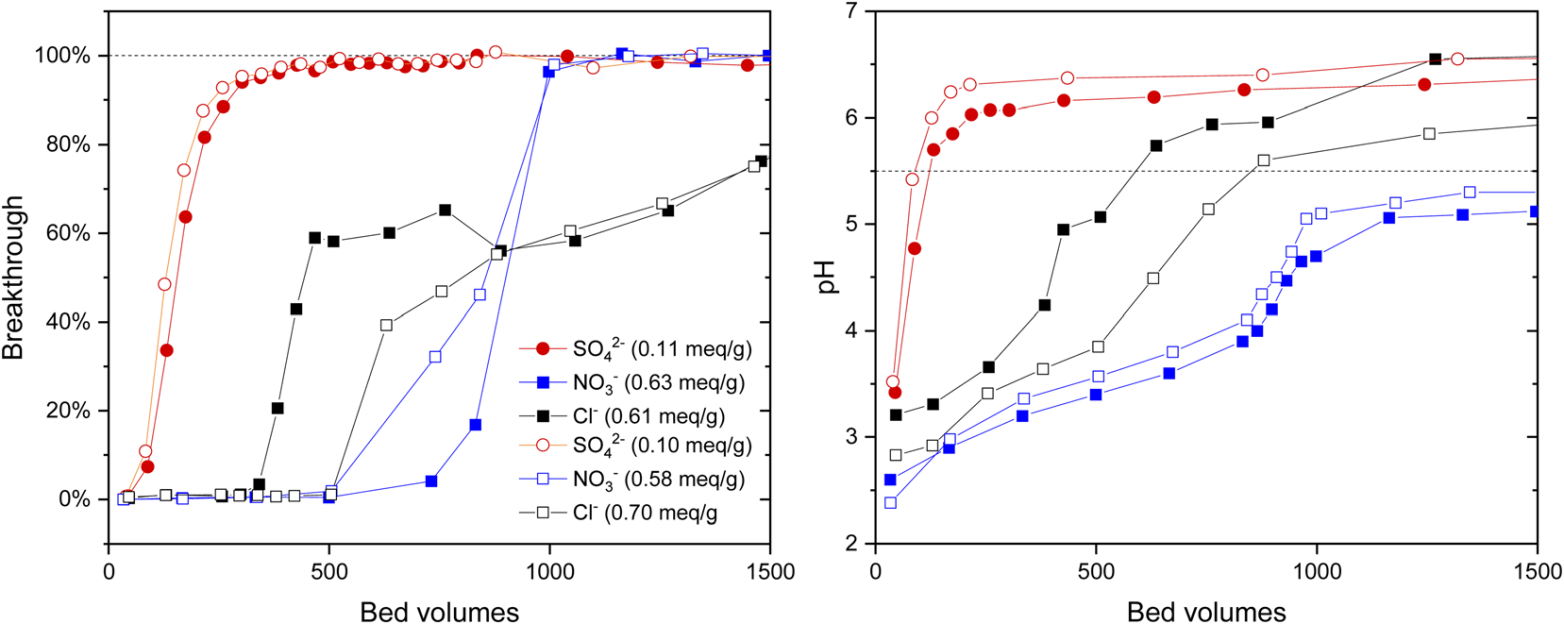
Effect of SO_4_^2−^ and NO_3_^−^ anions (10 mM) to uptake of IO_3_^−^ (*c* = 1 mM) on ZrO_2_ (open symbols) and Zr(Sb)O_2_ (filled symbols) and corresponding pH curves. The uncertainties are not shown in the graph due to the clarity but were below 2.5% for the determined apparent capacities.

The uptake of IO_3_^−^ (1 mM) from solutions of competing anions (SO_4_^2−^, Cl^−^ and NO_3_^−^) at ten times higher concentration (10 mM) was studied in columns. The BT-curves were rather similar between the two materials (left side graph in Fig. 4). These three anions had significantly different effects on the IO_3_^−^ uptake profile and apparent capacity. 100% BT was reached first in SO_4_^2−^ bearing solution, just after 250 BV's corresponding to 0.11 and 0.10 mmol g^−1^ IO_3_^−^ apparent capacity for Zr(Sb)O_2_ and ZrO_2_, respectively. Similar symmetrical BT was observed in NO_3_^−^ solution, although 100% BT was observed after 600 BV's (0.63 and 0.58 mmol g^−1^). One possible explanation for this would be the divalent charge of SO_4_^2−^ compared with the monovalent charge of NO_3_^−^. However, in Cl^−^ solution a totally different IO_3_^−^ uptake behavior was observed: the BT started at 250 BV's but exhibited differently shaped slope, and no complete BT was reached during the experiment which was stopped at >95% BT at about 2000 BV's (0.61 and 0.70 mmol g^−1^). The pH curves were different depending on the competing anion (right side graph in Fig. 4): the effluent pH stabilizes with SO_4_^2−^ already after 100 BV's, with NO_3_^−^ it lasts until 1000 BV's and with Cl^−^ until 500 BV's. Interestingly, this stabilization occurs at pH 5 with NO_3_^−^ that is lower than the feed solution pH (5.5), but with SO_4_^2−^ and Cl^−^ the pH stabilizes at higher pH at about 6.5 indicating the release of OH^−^ from the material into the solution.

Due to the strong interfering effect of SO_4_^2−^ and its relevance in decontamination environments, the uptake and competition between SO_4_^2−^ and IO_3_^−^ was further probed with the additional series of column experiments.

**Fig. 5:**
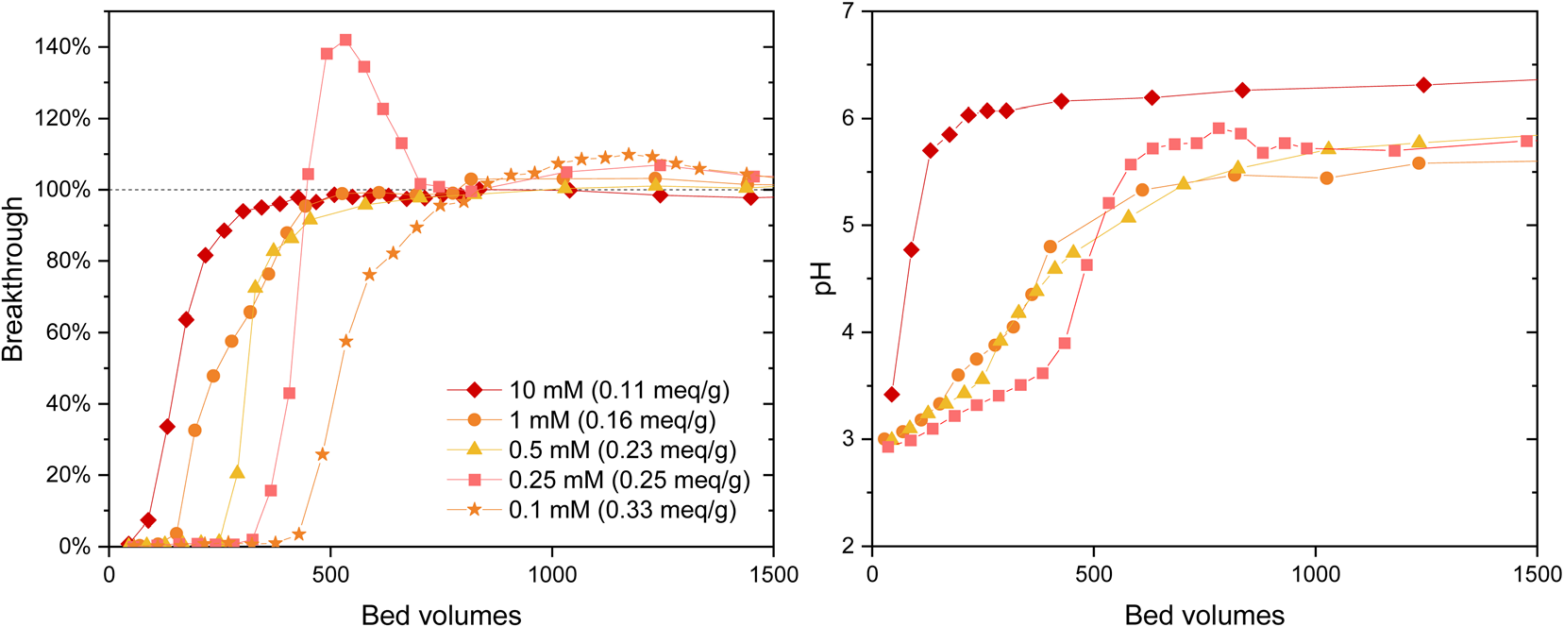
IO_3_^−^ (*c* = 1 mM) breakthrough and pH curves for Zr(Sb)O_2_ columns in different SO_4_^2−^ concentrations. The equilibrium uptake is represented in parentheses of each legend. The uncertainties are not shown in the graph due to the clarity but were below 4.0% for the determined apparent capacities.

**Fig. 6:**
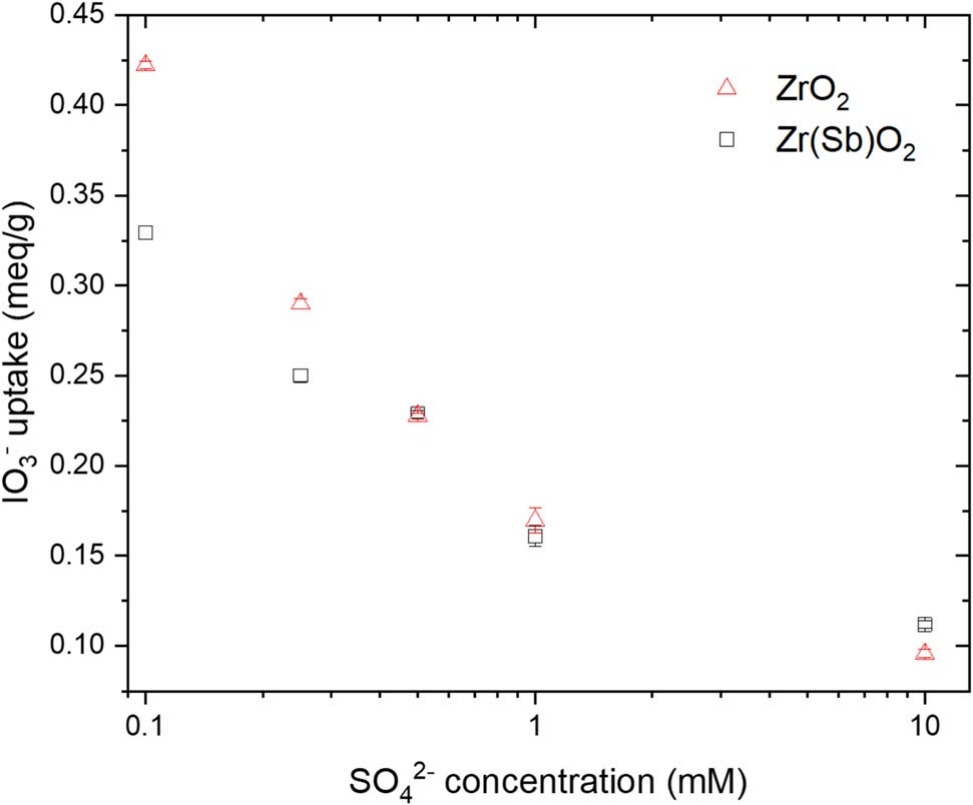
Effect of SO_4_^2−^ concentrations on IO_3_^−^ (*c* = 1 mM) uptake on ZrO_2_ and Zr(Sb)O_2_. Note the logarithmic scale on *x*-axis.

The IO_3_^−^ BT curves for Zr(Sb)O_2_ (Fig. 5) and ZrO_2_ (SI) show a logical trend with a rising SO_4_^2−^/IO_3_^−^ ratio: the BT reaches 100% earlier with the higher ratios. Regarding IO_3_^−^ uptake, a significant decrease in was observed when raising the SO_4_^2−^ concentration from 0.1 (*e.g.*, Zr(Sb)O_2_: 0.33 meq g^−1^ ± 1.10%) to 10 mM (0.11 meq g^−1^ ± 1.96%) (Fig. 6) and the effect was somewhat more profound with ZrO_2_ material. Although SO_4_^2−^ strongly competes with IO_3_^−^, even at SO_4_^2−^/IO_3_^−^ ratio of 100 the uptake of IO_3_^−^ is still about 0.1 meq g^−1^ which is about 20–30% of the maximum uptake in SO_4_^2−^ solution.

In 0.25 mM SO_4_^2−^ solution the IO_3_^−^ BT reaches above 100% and then stabilizes to 100% (Fig. 5). This experiment was repeated twice with nearly identical results (see ESI†). This is because in the early phase the adsorption sites were not in equilibrium with the solution composition, *i.e.*, more IO_3_^−^ was adsorbed compared with SO_4_^2−^ because of the deficiency of the latter. Later, when more SO_4_^2−^ was introduced to the column, some adsorbed IO_3_^−^ was released to the solution by SO_4_^2−^ and the equilibrium was reached. In the more dilute 0.1 mM solution similar behavior was also observed but to a milder extent: the BT reaches over 100% but not as considerably as with 0.25 mM SO_4_^2−^ solution. After the loading, IO_3_^−^ was desorbed from all columns using 0.1 M NaOH resulting in rather consistent complete desorption of the adsorbed IO_3_^−^.

#### 3.2.2 Column elution experiments

**Fig. 7:**
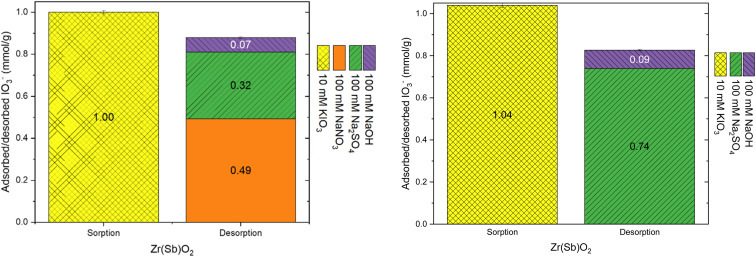
Uptake with 10 mM KIO_3_ solution and desorption with different solutions on Zr(Sb)O_2_.

**Fig. 8:**
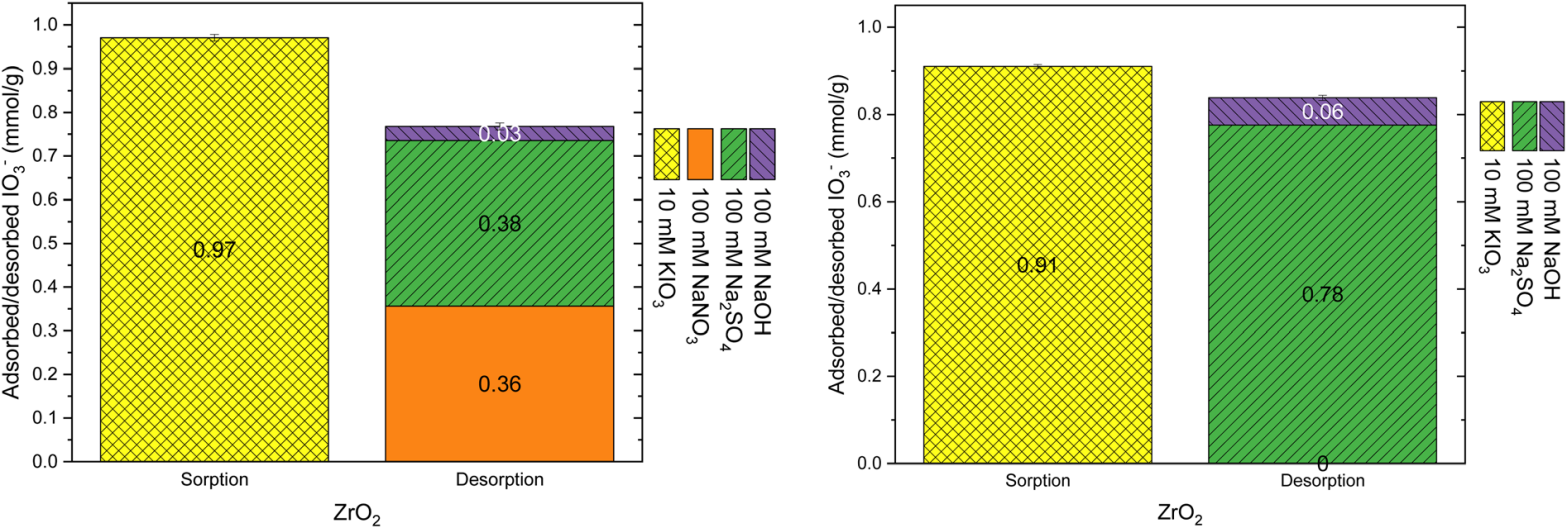
Uptake with 10 mM KIO_3_ solution and desorption with different solutions on ZrO_2_.

The elution properties of the materials were tested based on the observed selectivity preference of the anions (NO_3_^−^ < SO_4_^2−^ < OH^−^) at higher concentration (100 mM). The consecutive elutions with NaNO_3_, Na_2_SO_4_ and NaOH desorbed 50%, 32% and 7%, respectively of the adsorbed IO_3_^−^ (1.02 ± 0.02 mmol g^−1^) from the Zr(Sb)O_2_ material (left side graph in Fig. 7). The remaining 12% of IO_3_^−^ was left bound to the material which was qualitatively confirmed with gamma measurement (ESI†) indicating that IO_3_^−^ remained adsorbed and was not *e.g.*, evaporated as I_2_ due to redox reactions. With the ZrO_2_ material, the corresponding desorption percentages were 37%, 39% and 3%, and 20% of IO_3_^−^ remained adsorbed to the material (left-side graph in Fig. 8). The remaining IO_3_^−^ could not be desorbed even with NaOH which contradicted the earlier desorption experiments (see 3.2.1 Column load and desorption experiments) where constant 100% desorption was observed for almost all the columns. The difference between these experiments was in the IO_3_^−^ loading solution: here pure 10 mM KIO_3_ solution was used whereas in the earlier experiments loading was done from 1 mM KIO_3_ with the varying concentration of SO_4_^2−^. A plausible explanation could be that with higher IO_3_^−^ concentration and without any competing anions, a fraction of IO_3_^−^ could react with zirconium oxide surface resulting in irreversible sorption (inner-sphere complexation). The competing SO_4_^2−^ ions would most probably also compete for these irreversible binding sites and perhaps even with higher preference. The repetition of the experiment with only Na_2_SO_4_ and NaOH as desorption agents gave similar results as the elution with three solutions in sequence (right side graphs in Fig. 7 and 8) *i.e.* the IO_3_^−^ fraction eluted with SO_4_^2−^ was similar with what was eluted with a consecutive combination of NO_3_^−^ and SO_4_^2−^. This suggests that NO_3_^−^ competes for the anion exchange sites of the material but SO_4_^2−^ also competes with IO_3_^−^ for other binding sites and the preferences between these sites are different. The exchangeable anion capacity of both materials was about 0.8–0.9 meq g^−1^ which is well in line with the values reported in literature^23,37,38^ and in other experiments within this study.

#### 3.2.3 Regeneration experiments in column

**Fig. 9:**
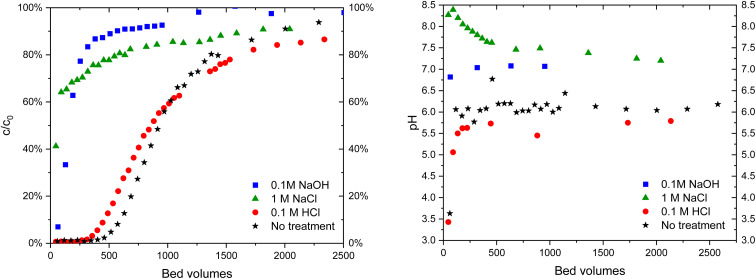
IO_3_^−^ (*c* = 10 μM) breakthrough in 10 mM Na_2_SO_4_ solution (left) in Zr(Sb)O_2_ columns of untreated material and after treatments with different solutions and the corresponding pH curves (right).

**Fig. 10:**
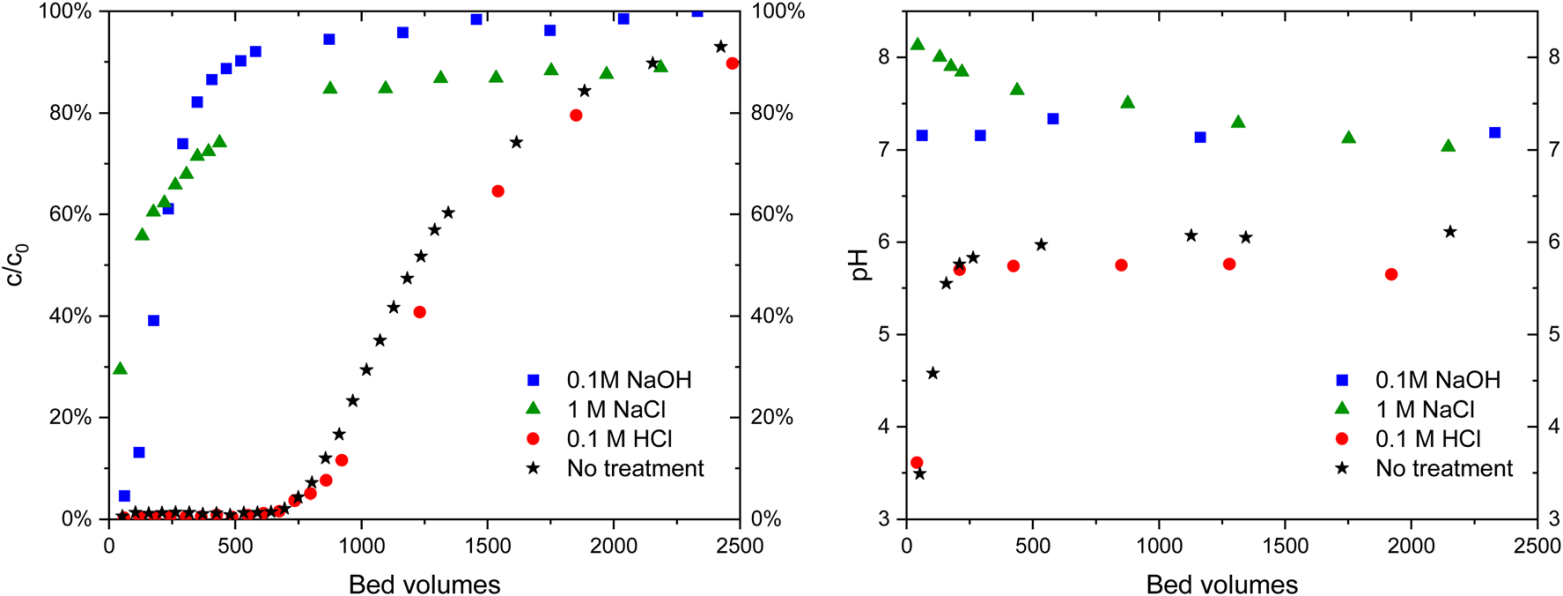
IO_3_^−^ (*c* = 10 μM) breakthrough in 10 mM Na_2_SO_4_ solution (left) in ZrO_2_ columns of untreated material and after treatments with different solutions and corresponding pH curves (right).

The regeneration of the materials was studied with 0.1 M NaOH, 1 M NaCl and 0.1 M HCl solutions (Zr(Sb)O_2_: Fig. 9, ZrO_2_: Fig. 10). With non-treated Zr(Sb)O_2_ the uptake of IO_3_^−^ (10 μM) in a high excess of SO_4_^2−^ (10 mM) was 7.54 ± 0.03 μeq g^−1^. HCl regenerated the materials efficiently as the total IO_3_^−^ uptake was even increased to 10.7 ± 0.1 μeq g^−1^. Instead, with NaCl (3.63 ± 0.04 μeq g^−1^) and NaOH (1.82 ± 0.03 μeq g^−1^) only partial regeneration was achieved. With ZrO_2_, similar results were observed (ESI†). In addition, only HCl was able to regenerate the pH lowering capability of the material (right side graphs in Fig. 9 and 10). After the treatment with 1 M NaCl, the pH rose from 6 to about 8 which indicates a release of OH^−^ from the material. However, it remains unexplained why the pH rose less (from 6 to 7) after 0.1 M NaOH treatment.

The regeneration behavior is explained by ion exchange: after the treatment with NaOH the material is in OH^−^ form:

3 Na^+^ + OH^−^ + Zr – OH_2_^+^IO_3_^−^ ⇌ Na^+^ + IO_3_^−^ + Zr – OH_2_^+^OH^−^

Without any further treatment, IO_3_^−^ is not able to exchange with OH^−^ in the material to the same extent as with the fresh material. The treatment with 0.1 M HCl returns the material to Cl^−^ form:

4 H_3_O^+^ + Cl^−^ + Zr – OH_2_^+^OH^−^ ⇌ 2H_2_O + Zr – OH_2_^+^Cl^−^

The same applies most probably to 1 M NaCl solution as well, but the conversion is not complete at neutral pH.

**Fig. 11:**
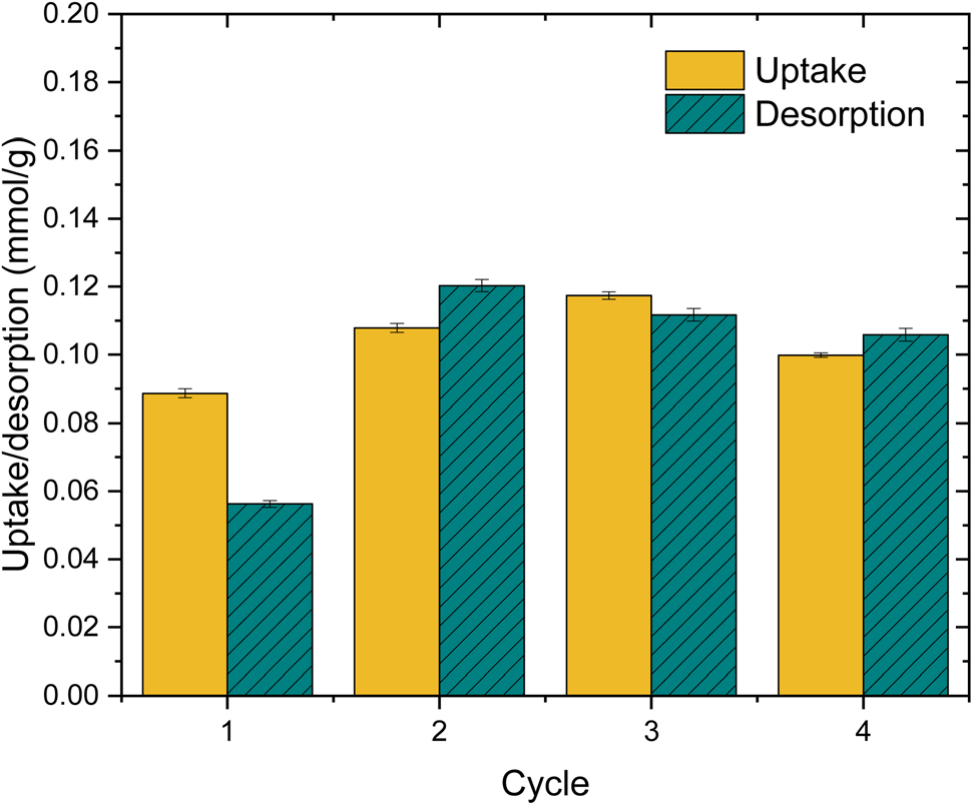
A total uptake of Zr(Sb)O_2_ after the several successive load (10 mM Na_2_SO_4_, 1 mM KIO_3_ pH 6), desorption (0.1 M NaOH) and regeneration (0.1 m HCl) cycles.

The practical regeneration of Zr(Sb)O_2_ material was tested with four IO_3_^−^ uptake/eluent cycles using 0.1 M NaOH as an eluent and 0.1 M HCl for the regeneration of the material between the cycles (Fig. 11). The regeneration efficiency remained high for all the cycles and the IO_3_^−^ uptake was approximately 0.10 ± 0.01 meq g^−1^ (the uptake and elution curves in ESI†). The successive cycles showed some variation in the eluted IO_3_^−^ fraction and the largest deviation was associated to the first cycle where non-treated material was used. This resulted in the lower IO_3_^−^ uptake and the lower elution percentage compared with HCl regenerated material at cycles 2 to 4, due to unexchangeable (inner-sphere complexation) IO_3_^−^ uptake.

### 3.3. Solid sample characterization

#### 3.3.1 Specific surface area measurements

The specific surface areas of the materials were analysed with nitrogen adsorption–desorption (Table 2). Zr(Sb)O_2_ exhibited slightly larger specific surface area compared with ZrO_2_. That was expected due to the disorder caused by a guest atom, Sb, in the ZrO_2_ structure. In general, Zr(Sb)O_2_ had higher maximum IO_3_^−^ uptake in the uptake experiments compared with ZrO_2_, but much less than the difference in the surface areas. Most probably the surface area is not significant attribute in the anion exchange behaviour of the material on this scale.

**Table 2:** Specific surface area, pore volume and pore diameter of the materials

	*A*_BET_ m^2^ g^−1^	*V*_total_ cm^3^ g^−1^	*D*_pore_ nm
Zr(Sb)O_2_	137 ± 2	0.07	2.5
ZrO_2_	96 ± 3	0.03	3.0

#### 3.3.2 I and Sb K-edge XANES

**Fig. 12:**
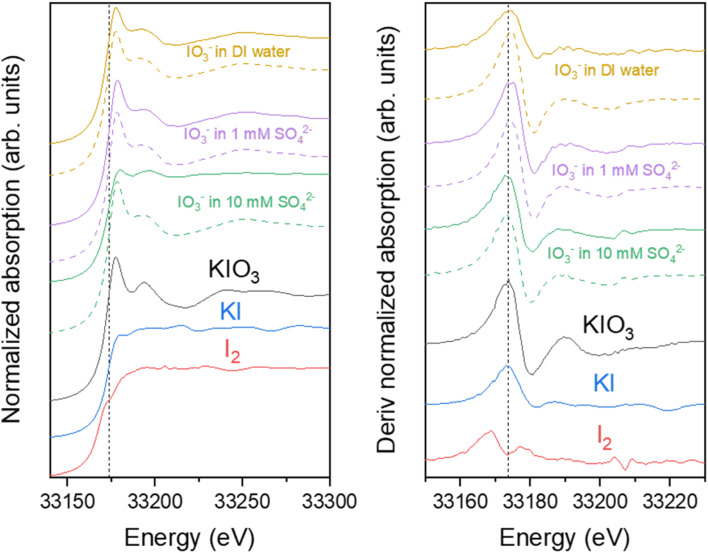
Left: I K-edge XANES spectrum of Zr(Sb)O_2_ (solid line) and ZrO_2_ (dashed line) loaded with IO_3_^−^ in deionized (DI) water and different sulfate concentrations along spectra of the KIO_3_, KI and I_2_ references. The vertical dashed line shows the position of iodate main absorption peak, as known as the first derivate maximum of the KIO_3_ reference. Right: The corresponding first derivative relatively to energy spectra. All spectra were vertically shifted for clarity.

I K-edge XANES spectra of the IO_3_^−^ loaded materials were measured to determine iodine oxidation state after adsorption (Fig. 12). The iodine K-edge XANES spectra of all the samples show similar strongly characteristic shape of IO_3_^−^, with some slight differences around the white line due to small changes in average geometry around iodine, except for the Zr(Sb)O_2_ in the most concentrated 10 mM SO_4_^2−^solution. In the latter case, the IO_3_^−^ spectral features were still visible but the overall spectrum was flattened indicating the partial reduction of iodine by comparison to KI and I_2_ reference spectra. The reduction of IO_3_^−^ to I^−^ was observed only for the Zr(Sb)O_2_ sample from the concentrated SO_4_^2−^ solution, which should not be redox active as such. However, in column experiments it was observed that SO_4_^2−^ considerably lowers the total uptake of IO_3_^−^. The most probable reason why the reduction of IO_3_^−^ was not observed in the samples with dilute SO_4_^2−^ is that in those samples the high excess of IO_3_^−^ masks the contribution of I^−^ to the XANES spectrum. In concentrated SO_4_^2−^ solution, IO_3_^−^ is adsorbed less which makes the relative concentration of I^−^ higher, thus making it visible in the spectrum. This would also indicate that SO_4_^2−^ is not preventing this redox dependant adsorption mechanism effectively, which is logical as the reduction of S(vi) by Sb(iii) is not energetically favoured.

**Fig. 13:**
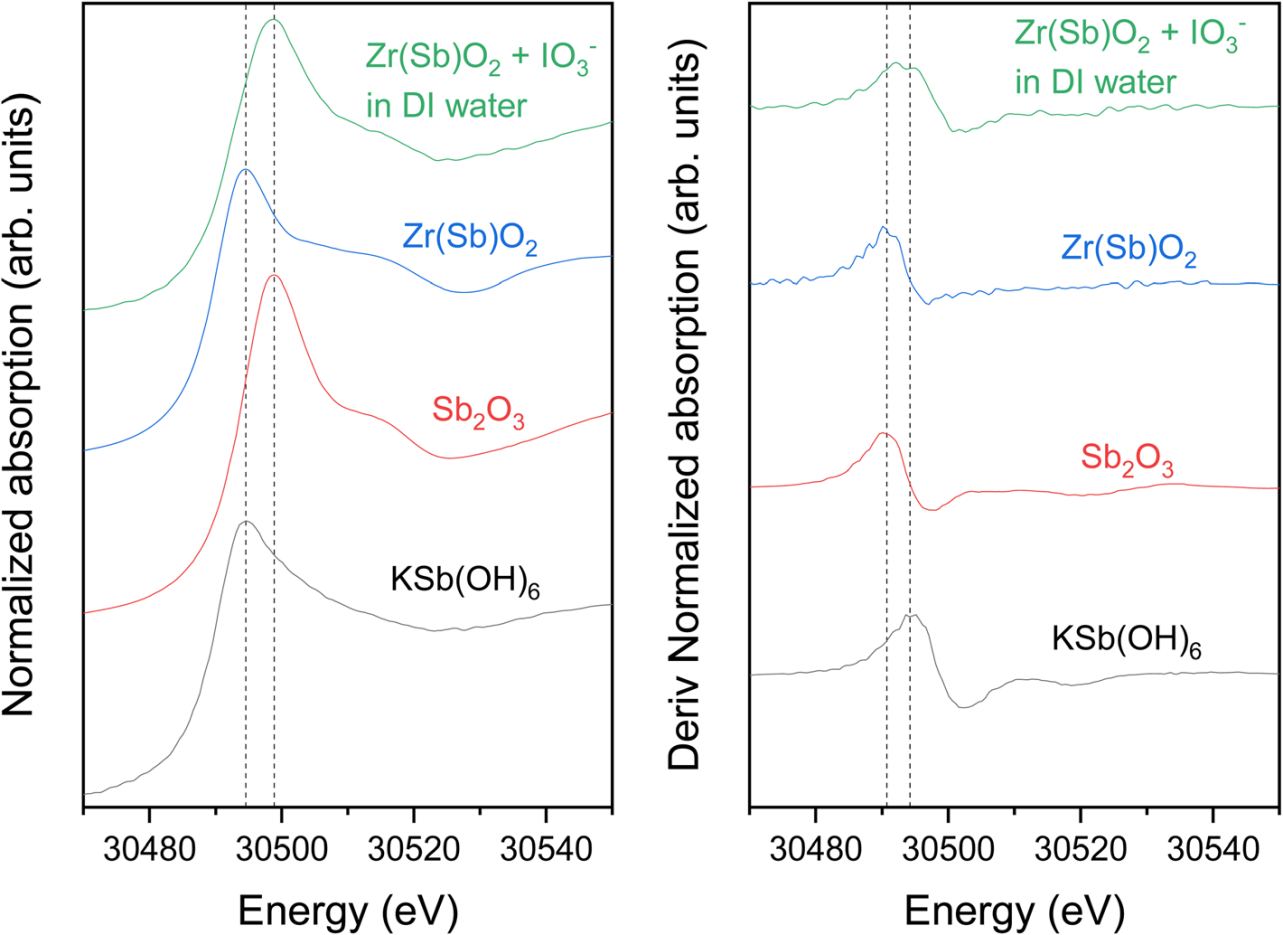
Sb K-edge XANES spectra of Zr(Sb)O_2_ samples (as prepared and 3.6 mmol g^−1^ IO_3_^−^ loaded) compared with Sb_2_O_3_ and KSb(OH)_6_ references. The dashed vertical lines show the absorption peak location of Sb(iii) and Sb(v).

The Sb K-edge XANES spectra were measured to see if Sb oxidation state changes in the material during the uptake process (Fig. 13). Sb remained as Sb(iii) after the Zr(Sb)O_2_ synthesis, but after the contact with IO_3_^−^ solution it partly oxidizes to Sb(v) as seen on the derivative spectra (Fig. 13) which is showing a bimodal white-line. However, the possible oxidation by dissolved oxygen needs to be considered. Since reduction of I was observed in I K-edge XANES, it seems highly probable that Sb is the reason for this. In total context, this must be a secondary adsorption mechanism as 1 gram of 5 molar-% Zr(Sb)O_2_ could reduce theoretically only 0.04 mmol of IO_3_^−^ to I^−^ and the fore-mentioned competing oxidation by dissolved oxygen most likely even lowers this value.

#### 3.3.3 I, and Zr K-edge EXAFS

**Fig. 14:**
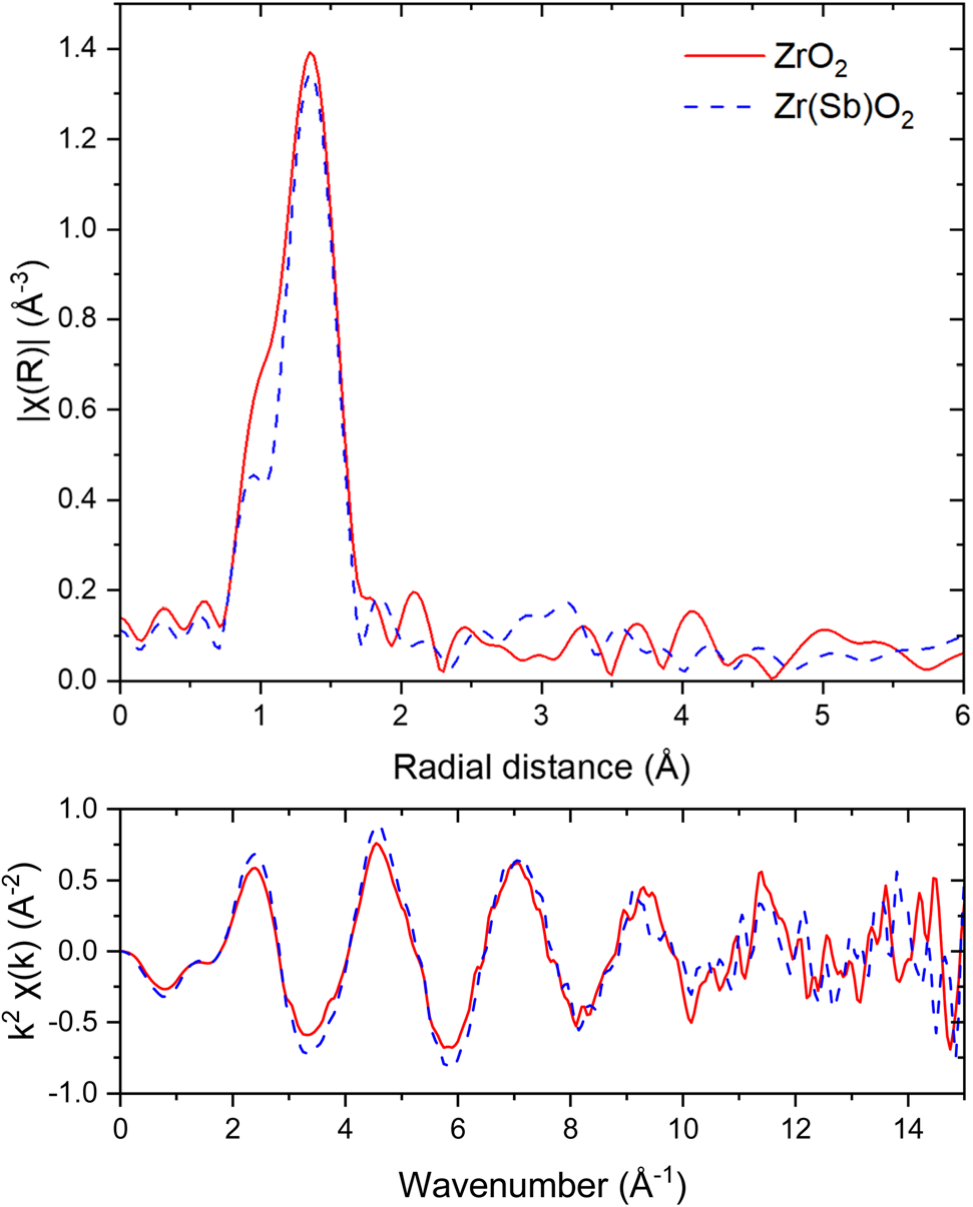
I K-edge EXAFS in *R*-space (upper graph) and *k*-space (lower graph) spectra of ZrO_2_ (solid red lines) and Zr(Sb)O_2_ (dashed blue lines) after IO_3_^−^ loading. FT window of 3 to 13.3 Å^−1^ was used.

No significant difference for either material before and after IO_3_^−^ adsorption was observed, in either Zr or I K-edge EXAFS. The Fourier transforms of I K-edge EXAFS oscillations of adsorbed IO_3_^−^ (Fig. 14) showed a strong peak centred approximately at 1.4 Å which is related to three oxygen atoms covalently bound to iodine in the molecule.^39^ Outside the oxygens, nothing significant was observed. This indicates that there is no close Zr neighbour for iodine. However, the possibility of heterogeneous distribution of local environments cannot be excluded. The EXAFS spectra of the materials with the adsorbed iodine were fitted (ESI†) with a simple first O shell path using Artemis, assuming I–O distance of 1.8 Å and coordination number of 3. The optimized Δ*E*_0_ was slightly elevated (15.24 ± 0.04 eV) for an unknown reason but also previously published values, even for reference materials, like KIO_3_, have been relatively high (10 eV).^39^ The fits including the closest oxygens reproduced the experimental data sufficiently well regarding the simplicity of the fitting approach (*R*-factor 0.0102). Only for Zr(Sb)O_2_ with the highest (10 mM) SO_4_^2−^ the fitting was not successful, because of partial reduction of IO^3−^ observed in XANES measurements. Fitting showed constant coordination number (3) and distance (1.8 Å) but the Debye–Waller factor showed a trend where the ZrO_2_ sample with the highest IO_3_^−^ loading (in DI water) showed also the highest degree of disorder (*σ*^2^ = 0.0027 ± 0.0007) and the sample with the lower loading had decreasing disorder with rising SO_4_^2−^ concentration (1 mM: 0.0022 ± 0.0005; 10 mM 0.0015 ± 0.0006). This could be explained by the more homogeneous adsorption site distribution in the conditions with high concentration of competing anions. With Zr(Sb)O_2_ samples, the other fitting parameters were similar with the pure ZrO_2_ although the disorder was increasing with decreasing IO_3_^−^ loading. This is most probably explained by the increasing significance of the partial reduction of IO_3_^−^ during the adsorption in the conditions with excess of competing SO_4_^2−^.

In Zr K-edge EXAFS spectra the signal at about 1.5 Å is assigned to the 1st shell oxygen and the second peak at 3.0 Å to 2nd shell Zr atoms (ESI†). These values correspond well to the values found in literature.^40,41^ Similar fitting as with I EXAFS was done for Zr. However, in all the samples no significant differences were found between the pristine and loaded ZrO_2_/Zr(Sb)O_2_ regarding the local coordination environment of Zr. Sb K-edge EXAFS spectra exhibited peaks at about 1.5 Å but the first shell fits of Zr and Sb (ESI†) did not reveal any significant differences, except that *σ*^2^ was higher for pure Zr(Sb)O_2_ compared with the material after IO_3_^−^ loading.

#### 3.3.4 Thermo gravimetric analysis

**Fig. 15:**
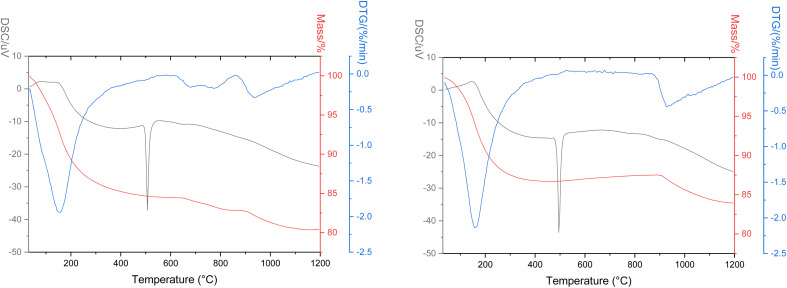
TGA curves of HCl and NaOH treated Zr(Sb)O_2_.

Before performing TGA, Zr(Sb)O_2_ was treated with 0.1 M NaOH or HCl in a column followed by washing with deionized water and drying in oven at 70 °C. In general, the TG data was similar for both NaOH and HCl treated material (Fig. 15, see ESI† for the GS-MS chromatograms). The phase transition from amorphous to tetragonal took place at about 500 °C which can be seen as an exothermic peak in DSC. The most significant mass loss was observed between 100 and 200 °C which was caused by the evaporation of adsorbed water confirmed by the collected MS-spectrum where a significant signal was observed at *m*/*z* 18. At the same temperature also notable signal from *m*/*z* 44 corresponding to CO_2_ was observed. It seems that the material is adsorbing significant amounts of CO_2_ from either or both air and solution like have been reported previously.^42^ The signal of CO_2_ was about two times higher for the NaOH treated material. In addition to the sharp CO_2_ signal at 100 to 200 °C, a broad CO_2_ signal was observed later at 600–1200 °C for the HCl treated material. At 500 °C, when the phase transition occurs, a sharp peak of CO_2_ was also observed. The most significant difference between the materials was observed in *m*/*z* 36 and 38 which corresponds to HCl with different Cl isotopes (^35^Cl: 75.77% ^37^C: 24.23%). The HCl treated material released a high amount of HCl at 700 °C with a sharp rise in the signal. NaOH treated material did not release HCl before the very end of the measurement where heating was already stopped. This suggests two different sites for Cl^−^ in the material: the first released at 700 °C originates from the ion exchange in the column whereas the later released tracks down to the synthesis. The previous has a much higher signal and the latter is only released after the structure starts to transform to a monoclinic structure. For the HCl treated material also SbCl_5_ was detected after the HCl release. This could be caused by the reaction of released HCl with the structural Sb.

### 3.4. Consideration on IO_3_^−^ adsorption mechanism and competition of other anions

The adsorption of IO_3_^−^ is strongly associated with the pH in the solution and indications on the sorption process can be drawn from the pH changes during the uptake. Firstly, the surface charge of the adsorbent materials changes according to the solution pH, which strongly affects their affinity to anions in the solution. The anion exchange on zirconium oxides have been extensively studied earlier^24,25,37,43^ and in principle, the anion exchange site on zirconium oxide can be represented as:

5 ≡Zr – OH + H^+^ ⇌ ≡Zr – OH_2_^+^

where the protonated surface hydroxyls act as positively charged anion exchange sites.

In addition, the possible ligand exchange is described as:

6 ≡Zr – OH + X^−^ ⇌  ≡Zr − X + OH^−^

where anion X^−^, *e.g.*, sulphate or iodate, is exchanged with OH^−^ in the material structure.^23^

Secondly, the uptake of IO_3_^−^ (or SO_4_^2−^) itself affects the solution pH. Zirconium oxides synthesized within this study tend to lower pH even in deionized solutions, *e.g.*, in the column experiments pH was lowered from 5.6 to 2.5 at the beginning of the experiments. The mechanism of this pH change is related to the synthesis derived anions (Cl^−^ and NO_3_^−^ depending on the synthesis conditions), which remain in the structure due to the incomplete exchange with OH^−^ during the synthesis, *i.e.* the materials are initially partly in the OH^−^ and partly in the Cl^−^-form.^23^ In addition, the form of the material can be changed by regeneration with dilute acid like HCl that changes the material back to the Cl^−^-form. The exchange of the adsorbed anions with OH^−^ leads to the pH drop in the solution, like shown in eqn (1) earlier. In the presence of IO_3_^−^ (or SO_4_^2−^), the competing reaction takes place preventing the drop of pH:

7 Zr – OH_2_^+^Cl^−^ + IO_3_^−^ ⇌ Zr – OH_2_^+^IO_3_^−^ + Cl^−^

This explains why pH (*i.e.*, the concentration of OH^−^) and SO_4_^2−^ concentration are critical parameters regarding the IO_3_^−^ adsorption on zirconium oxides.^31^

This study demonstrates that both adsorption mechanisms (eqn (5) and (6)) contribute to the adsorption of IO_3_^−^, although the outer-sphere complexation (eqn (7)) is evidently the main mechanism of uptake as the IO_3_^−^ sorption was observed to be highly reversible and efficient and fast desorption was achieved with relatively dilute NaOH (100 mM). Also, the sorption capability was efficiently regenerated using dilute acid like HCl which changes the material back to the Cl^−^-form. In addition, certain fractions of adsorbed IO_3_^−^ were eluted efficiently by solutions containing NO_3_^−^ and SO_4_^2−^. However, NO_3_^−^ eluted less IO_3_^−^ compared with SO_4_^2−^, and even combined they were not able to elute all adsorbed IO_3_^−^ indicating that there are significant selectivity differences between the anions due to *e.g.*, structural properties of the exchange sites. In the high excess of SO_4_^2−^ (10 : 1 in concentration) the apparent capacity of IO_3_^−^ remained reasonably high (about 0.10 meq g^−1^) indicating a certain selectivity to the latter. The inner-sphere complexation (eqn (6)) of IO_3_^−^ can be regarded as a minor uptake mechanism and it was only observed in the relatively high concentration (10 mM) of IO_3_^−^ and without any competing anions. This IO_3_^−^ fraction remained bound to the materials even after successive elutions with NO_3_^−^, SO_4_^2−^ or NaOH.

## 4. Conclusion

Hydrous zirconium oxide materials ZrO_2_ and its antimony-doped Zr(Sb)O_2_ counterpart exhibited excellent IO_3_^−^ adsorption properties regarding apparent capacity (>0.6 meq g^−1^) and especially selectivity in high excess of competing anions, such as environmentally relevant SO_4_^2−^. The selectivity differences of zirconium oxides to different anions were observed, as NO_3_^−^, SO_4_^2−^ and OH^−^ seem to compete with IO_3_^−^ for different available adsorption sites (competition decreasing in order OH^−^ > SO_4_^2−^ > NO_3_^−^). The materials exhibited the highest IO_3_^−^ removal when changed to Cl^−^ form with dilute HCl (about 5 times higher apparent capacity compared with the OH^−^-form). The materials also showed constant uptake performance during three load-regeneration cycles when regenerated with dilute acid (0.1 M HCl) which demonstrates the potential feasibility of the material for practical applications regarding sustainability and financial perspectives.

Based on the easy regeneration in dilute conditions and fast uptake, the main mechanism of uptake was concluded to be the ion-exchange between IO_3_^−^ and anions *e.g.*, NO_3_^−^, Cl^−^ and OH^−^ forming the outer-sphere complex with the materials. In the XAS data no external neighboring atoms were observed in the Zr or I K-edge EXAFS. This supports the conclusion regarding the outer-sphere complexation, although certain precautions should be taken as the reason for this could also be the relatively low concentrations of exchangeable anions (*e.g.*, in 4% of total mass Cl^−^ -form) or the amorphous structure of the materials resulting in the homogeneous distribution of the local coordination environments of the adsorption sites. Ligand exchange (inner-sphere complexation) between IO_3_^−^ and surface OH^−^ was observed to take place as a minor secondary adsorption mechanism in conditions without competing anions. IO_3_^−^ is known to form both inner- and outer-sphere complexes with oxides and the dominating mechanism depends on the ionic strength and pH of the solution.^44^ It seems that at least in the case of zirconium oxides, the type of competing anion can affect the proportions of the available sites as well due to electivity differences. In the presence of Sb doping, also a redox reaction between Sb and I was discovered and confirmed by the XANES data, but the mechanism only contributes slightly (theoretical capacity 0.04 mmol g^−1^) to the overall IO_3_^−^ (maximum apparent capacity about 1 mmol g^−1^) uptake. It can, however, become significant in concentrated matrices.

Further studies would be required for the identification of the different ion exchange sites and what is the fundamental chemical or physical explanation for the selectivity of zirconium oxides to IO_3_^−^ and SO_4_^2−^. This knowledge could be utilized for the manipulation of the material structure during the synthesis to furthermore improve the IO_3_^−^ selectivity.

## Conflicts of interest

There are no conflicts of interest to declare.

## Supplementary Material

RA-013-D2RA06489H-s001
