# Supplementary material for: Anion exchange on hydrous zirconium oxide materials: application for selective iodate removal
Source: RSC Adv. 2023 Jan 4;13(2):948–62. doi: 10.1039/d2ra06489h (PMC9811356; doi:10.1039/d2ra06489h)
Supplement: RA-013-D2RA06489H-s001 [file RA-013-D2RA06489H-s001.pdf]

Electronic Supplementary Material (ESI) for RSC Advances.
This journal is © The Royal Society of Chemistry 2022

Supplementary information:

Anion exchange on hydrous zirconium oxide materials: application for selective iodate removal

V. Suorsa[1,*], M. Otaki[1], T. Suominen[1], J. Virkanen[2], H. Reijola[2], R. Bes[3,4] and R. Koivula[1]

[1] *Radiochemistry Unit, Department of Chemistry, University of Helsinki, A.I. Virtasen aukio 1, 00014 Helsinki, Finland*

[2] *Department of Geosciences and Geography, University of Helsinki, Gustaf Hällströmin katu 2, 00014 Helsinki, Finland*

[3] *Department of Physics, University of Helsinki, PO Box 64, FI-00014 Helsinki, Finland*

[4] *Helsinki Institute of Physics, PO Box 64, FI-00014 Helsinki, Finland*

* *Corresponding author. E-mail address: valtteri.suorsa@helsinki.fi; ORCID: 0000-0003-1544-5283*

Abstract

The radioactive ^{129}I is a top-priority radionuclide due to its the long half-life (1.57×10^7 y) and high mobility. Because of the planned and accidental releases to the environment, specific separation technologies are required to limit the potential radiation dose to human beings. Zirconium oxides are known for their adsorption capability and selectivity to oxyanions and here the applicability to selective IO_3^- removal has been investigated regarding the uptake mechanism, regeneration and competition caused by other anions, like environmentally relevant SO_4^{2-}. Granular aggregates of hydrous zirconium oxides with and without Sb doping showed high potential for the selective IO_3^- removal in the presence of competing anions, like the fore mentioned SO_4^{2-} (apparent capacity between 0.1 – 0.4 meq/g depending on SO_4^{2-} concentration). The main uptake mechanism was found to be outer-sphere complexation (ion-exchange) to the protonated hydroxyl groups of hydrous zirconium oxides but also minor mechanisms were identified including inner-sphere complexation and reduction to I^-. The materials were observed to be easily and successively

regenerated using dilute acid. Hydrous zirconium oxides showed high potential for IO_3^- removal from waste solutions regarding technical (high selectivity and apparent capacity) and ecological/economic (feasible regeneration) aspects.

1. Equations for the capacity calculatios

The apparent capacity q of analyte by ion exchanger for a single sample is calculated by Equation (SI1):

$$q = \frac{(c_i - c_f) \times V}{m} \qquad \text{(Equation SI1)}$$

where

c_i = initial concentration of analyte in the solution.

c_f = final concentration of analyte in the solution.

V = volume of the solution

m = mass of the dry ion exchanger

The corresponding uncertainty of uptake is calculated by Equation (SI2):

$$\Delta q = \sqrt{\left(\frac{V}{m} \times \Delta\, c_i\right)^2 + \left(\frac{V}{m} \times \Delta\, c_f\right)^2 + \left(\frac{(c_i - c_f)}{m} \times \Delta V\right)^2} \qquad \text{(Equation SI2)}$$

Equation (SI2) does not take the errors of mass (0.05 %) into account as it is insignificant compared to errors concentration measurements (relative errors (1 σ) 0.5 – 50 % depending on the count rate for γ-detector or 0.1 unit for pH detector).

The total q_{total} is the sum of q_i of the individual fractions described by Equation (SI3):

$$q_{total} = \sum_{i=1}^{n} q_i \qquad \text{(Equation SI3)}$$

The corresponding uncertainty of uptake is calculated by Equation (SI4):

$$\Delta q_{total} = \sqrt{\sum_{i=1}^{n} \Delta q_i^{\,2}} \qquad \text{(Equation SI4)}$$

2. B: Application on selective IO_3^- removal from waste solutions

Table 1 Comparison of $^{127}IO_3^-$ and $^{125}IO_3^-$ apparent capacities determined in two parallel column experiments (total iodate concentration: 1 mM).

Iodine isotope	Analysis method	Apparent capacity [mmol/g]	Feed pH
$^{125}IO_3^-$	γ-detector	0.42 ± 0.02	5.6
$^{127}IO_3^-$	HPLC-ICP-MS	0.44 ± 0.02	5.7

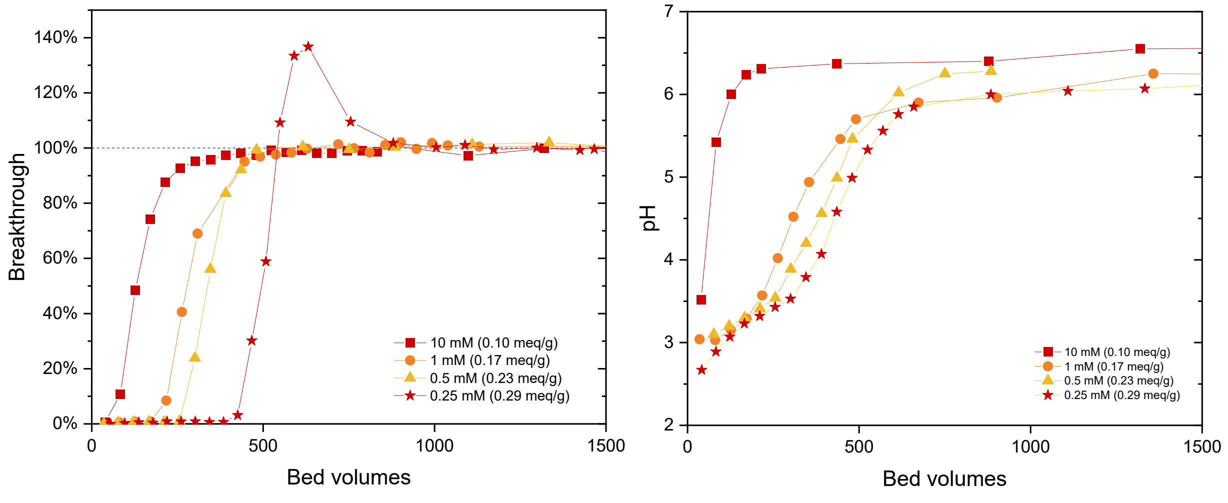

Figure 1 IO_3^- (c = 1 mM) breakthrough and pH curves for ZrO_2 columns in different SO_4^{2-} concentrations. The equilibrium uptake is represented in parentheses of each legend. The uncertainties are not shown in the graph due to the clarity but were below 4.0 % for the determined apparent capacities.

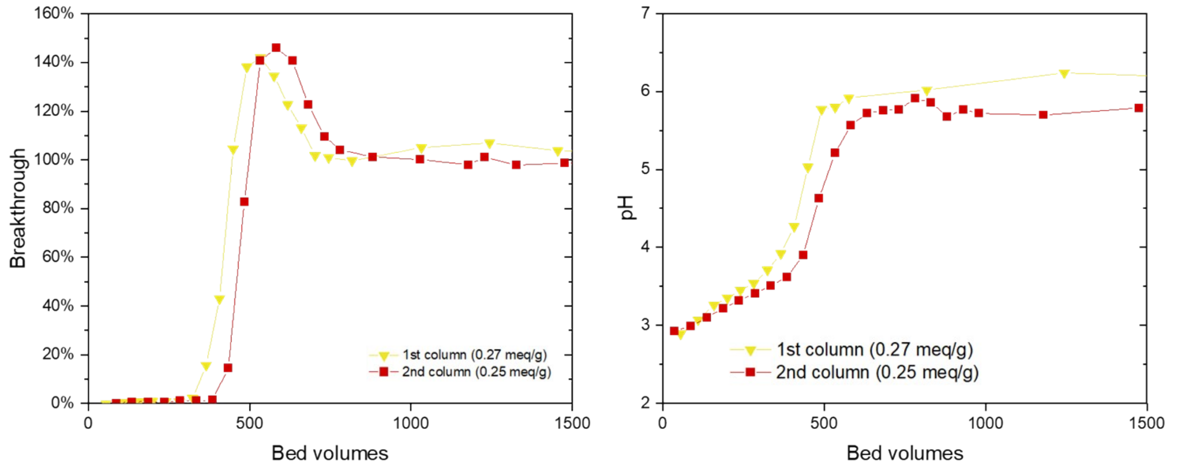

Figure 2 IO_3^- breakthrough and pH curves of two repetions of $ZrSbO_2$ columns in 0.25 mM SO_4^{2-} and 1 mM IO_3^-.

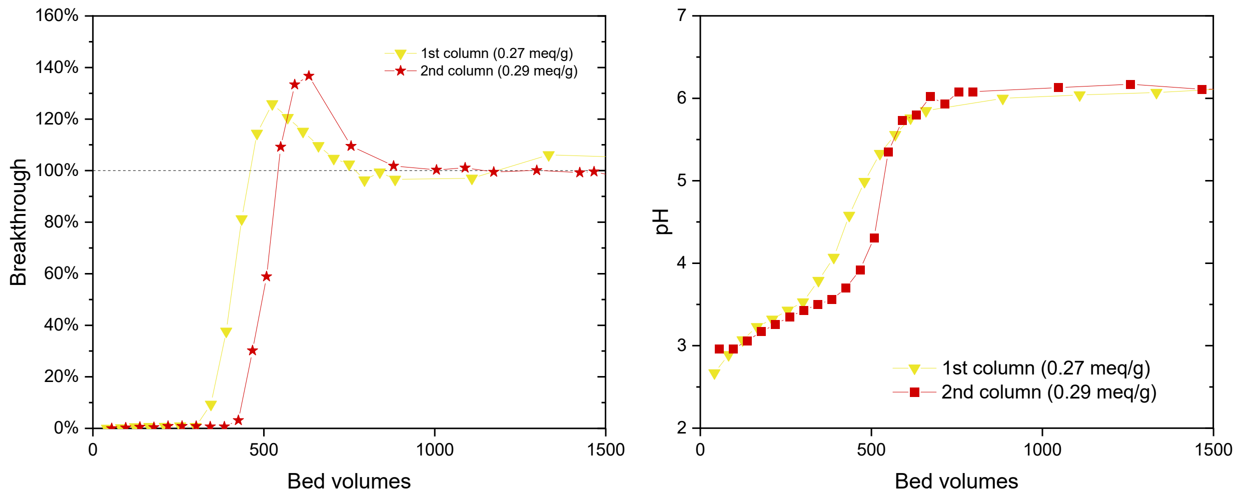

Figure 3 IO_3^- breakthrough and pH curves of two repetions of ZrO_2 columns in 0.25 mM SO_4^{2-} and 1 mM IO_3^-.

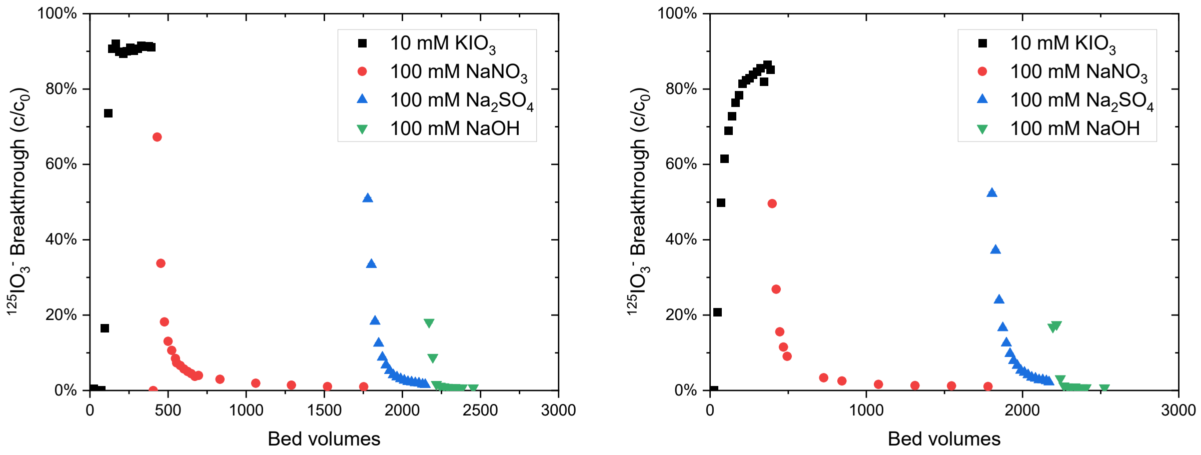

Figure 4 Column elution curves for different eluents for $Zr(Sb)O_2$ (left) and ZrO_2 (right) for the column elution experiment with $NaNO_3$, Na_2SO_4 and NaOH as eluents.

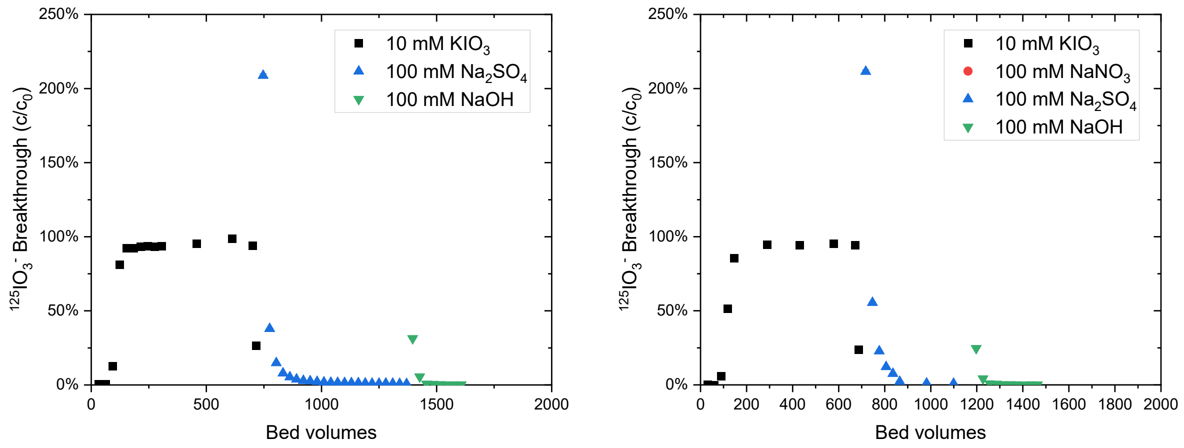

Figure 5 Column elution curves for different eluents for $Zr(Sb)O_2$ (left) and ZrO_2 (right) for the column elution experiment with Na_2SO_4 and NaOH as eluents.

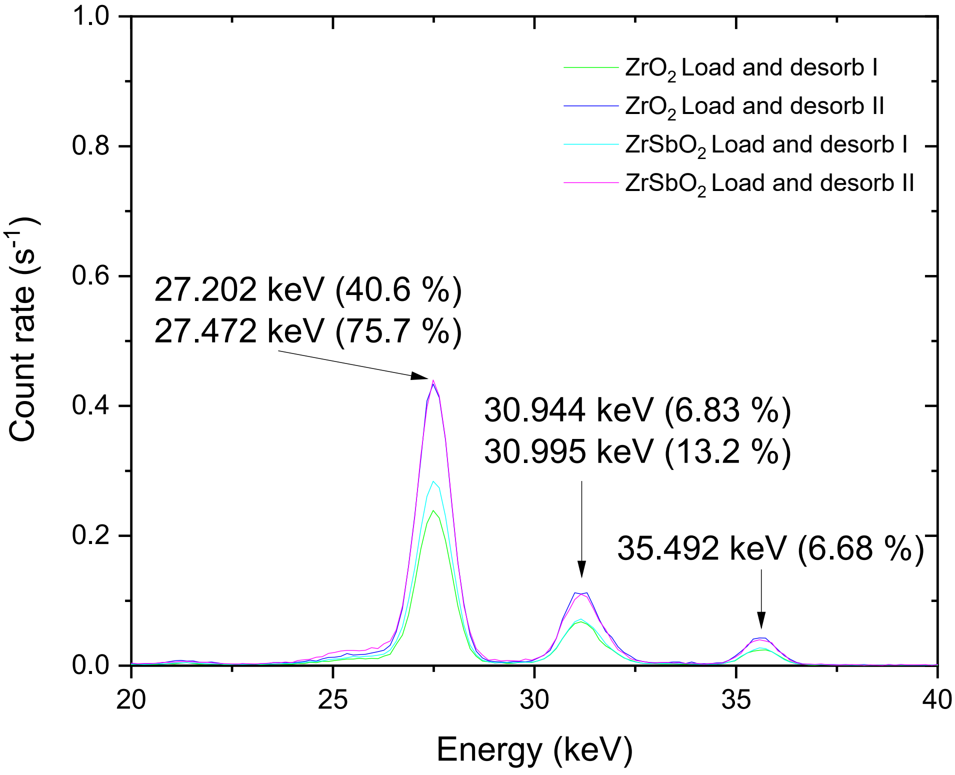

Figure 6 The gamma spectra of ^{125}I of the measured used column beds after the column elution experiments.

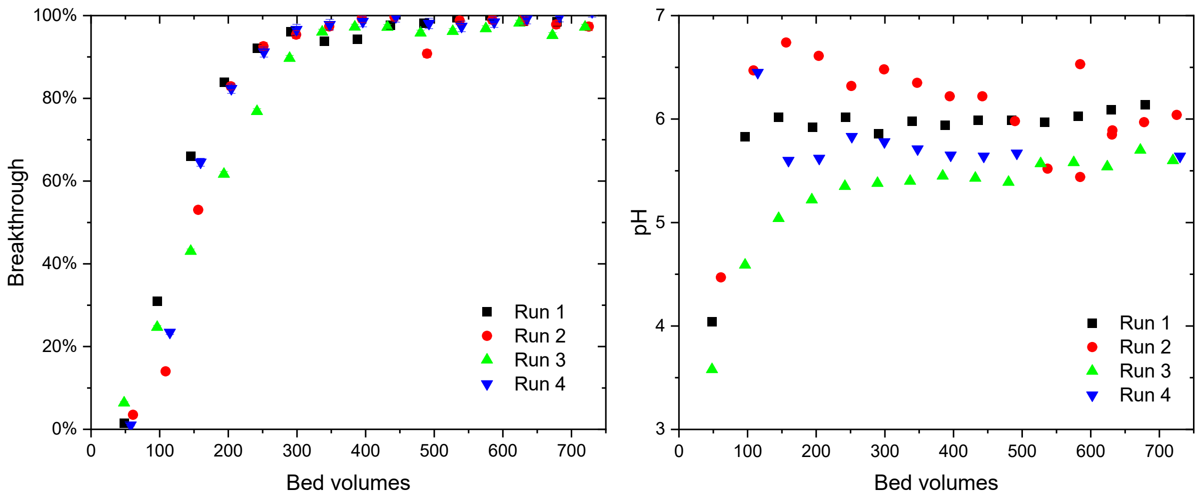

Figure 7 $^{125}IO_3^-$ breakthrough and corresponding pH curves as function of bed volumes for each load cycle in regeneration experiment with loading solution containing 10 mM SO_4^{2-} and 1 mM IO_3^-.

3. Solid sample characterization

3.1. XRD

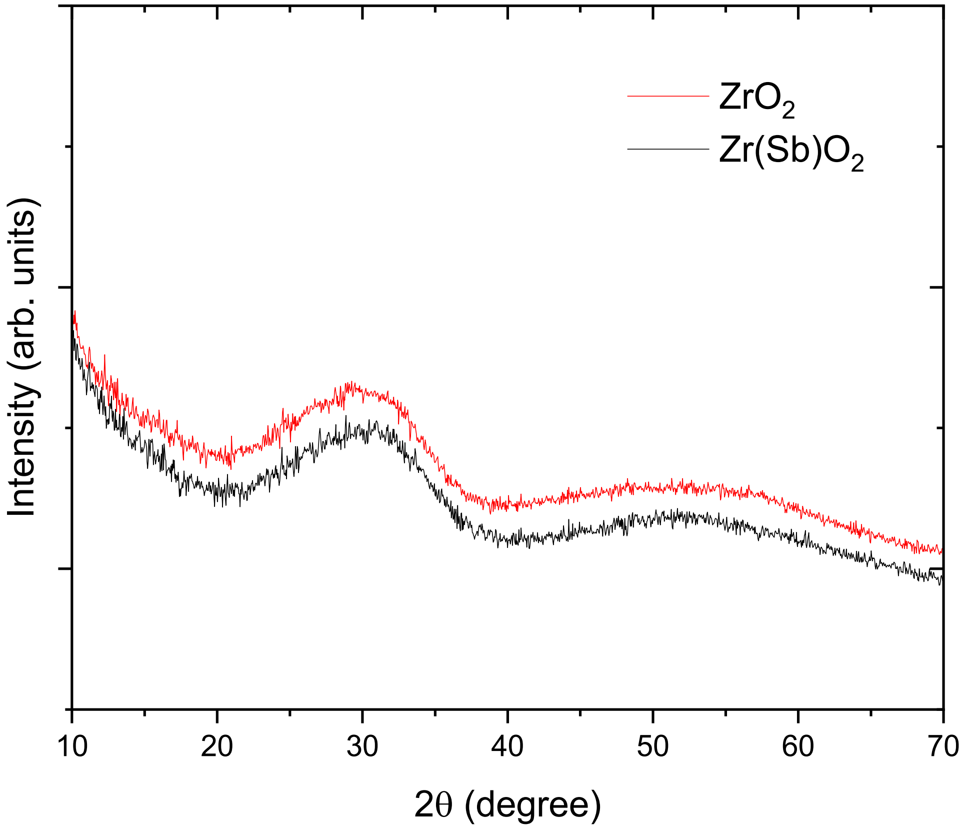

Figure 8 Powder XRD diffractograms of ZrO_2 and $Zr(Sb)O_2$.

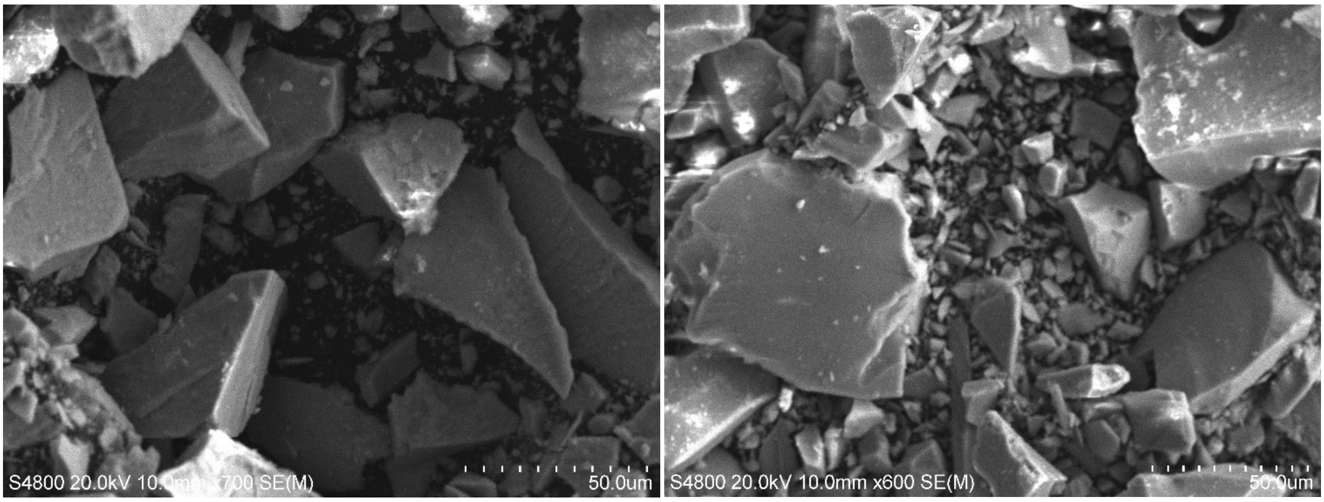

3.2. FE-SEM

3.3. EXAFS fittings

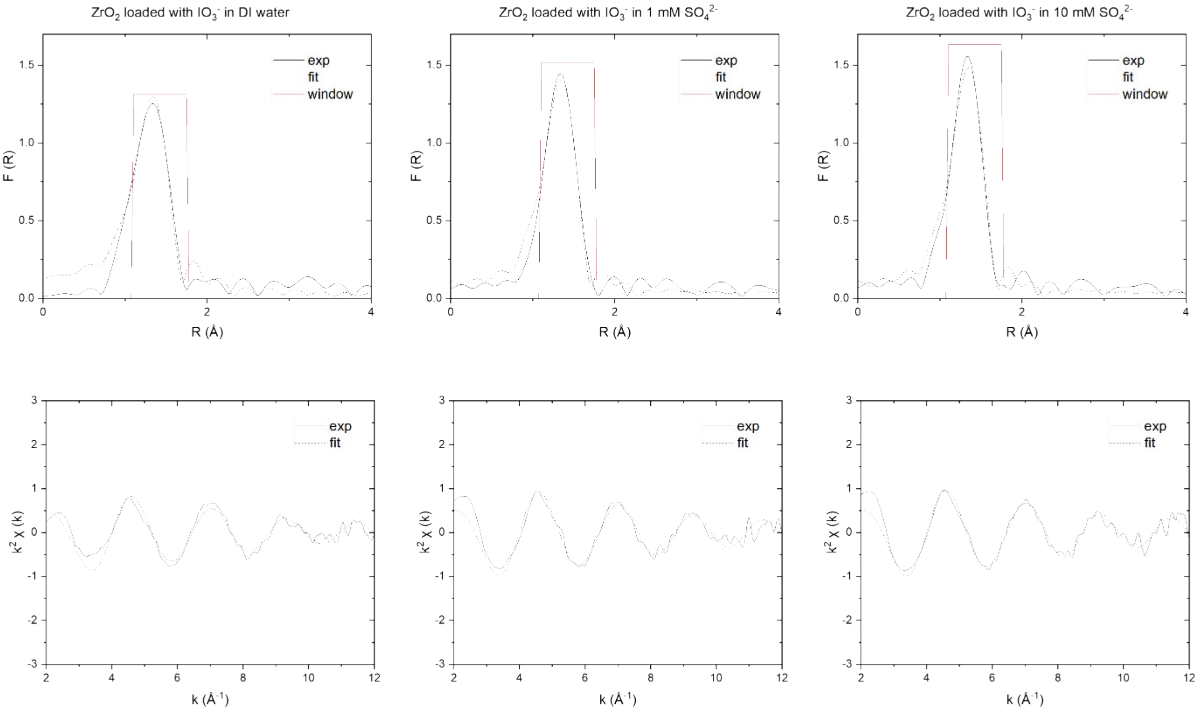

Figure 10 I K-edge EXAFS fits in k- and R-space for ZrO_2 samples.

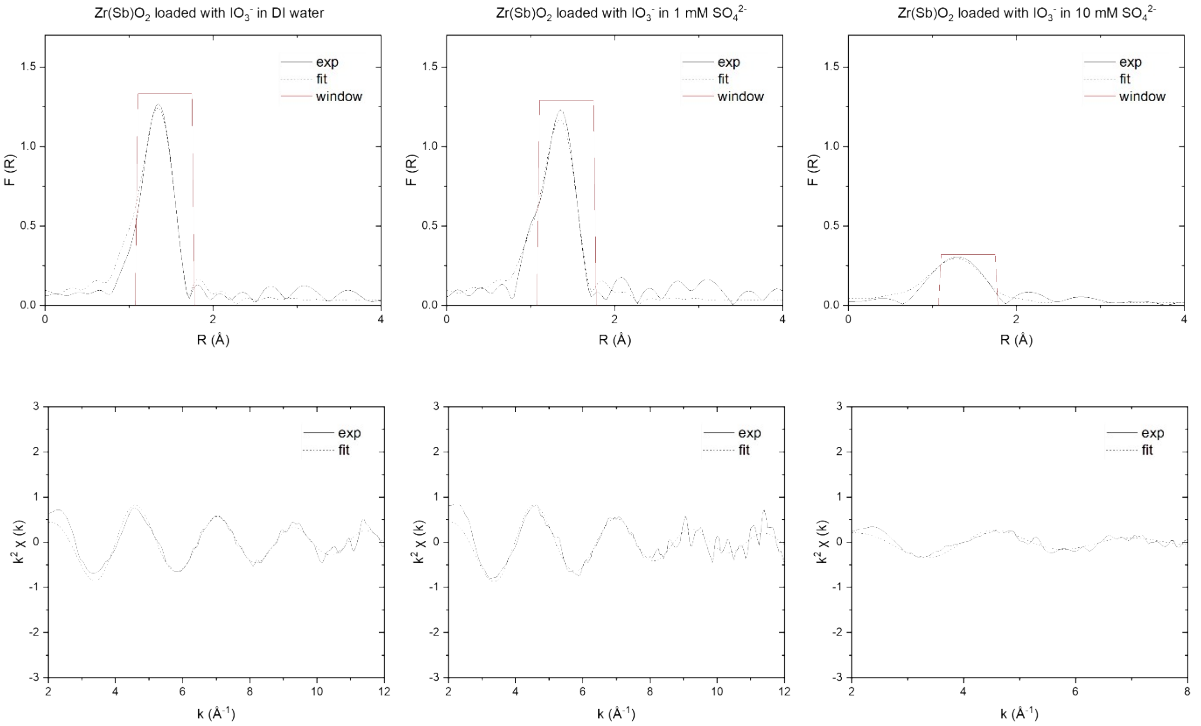

Iodine

Figure 11 I EXAFSf its of Zr(Sb)O2

Table 2 Fitting parameters for I K-edge EXAFS spectra measured for ZrO_2 and $Zr(Sb)O_2$ loaded with IO_3^- in different conditions. S_0^2 (0.98 ± 0.02) and ΔE_0 were extracted from the fit of $Zr(IO_3)_4$ reference sample and set as global parameters. The combined R-factor for the fit was 0.0097.

**Sample**	**ΔE**<br>**eV**	**R**<br>**Å**	**N**	**σ²**
**ZrO_2**				
DI water	15.2(1)	1.81(1)	3.0(2)	0.003(1)
1 mM SO_4^{2-}	15.2(1)	1.82(1)	3.3(1)	0.002(1)
10 mM SO_4^{2-}	15.2(1)	1.81(1)	3.3(2)	0.002(1)
**$Zr(Sb)O_2$**				
DI water	15.2(1)	1.81(1)	2.9(1)	0.002(1)
1 mM SO_4^{2-}	15.2(1)	1.82(1)	3.1(3)	0.003(2)
10 mM SO_4^{2-}	15.2(1)	1.83(2)	1.7(3)	0.014(3)

Zirconium

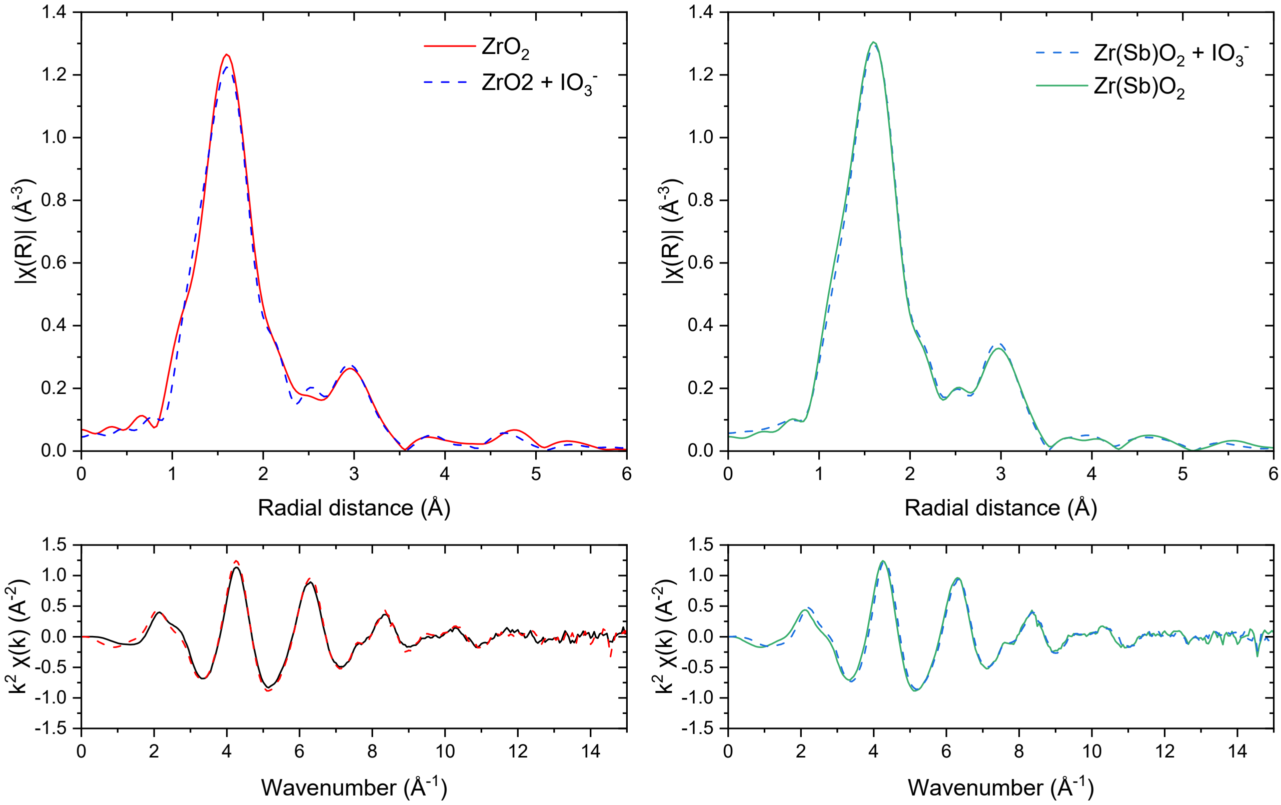

Figure 12 Zr K-edge EXAFS and Fourier transformed spectra of ZrO_2 (left graph) and $Zr(Sb)O_2$ (right graph) before and after IO_3^- adsorption. FT window of 3 to 12 $Å^{-1}$ was used.

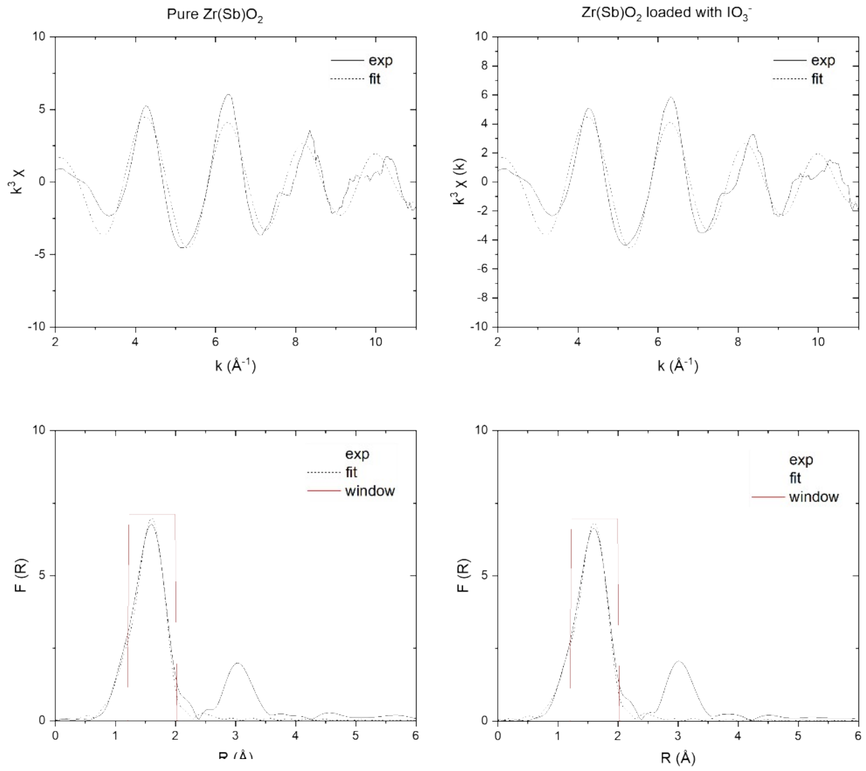

Figure 13 Zr K-edge EXAFS fits in R- and k-space for pure and loaded $Zr(Sb)O_2$.

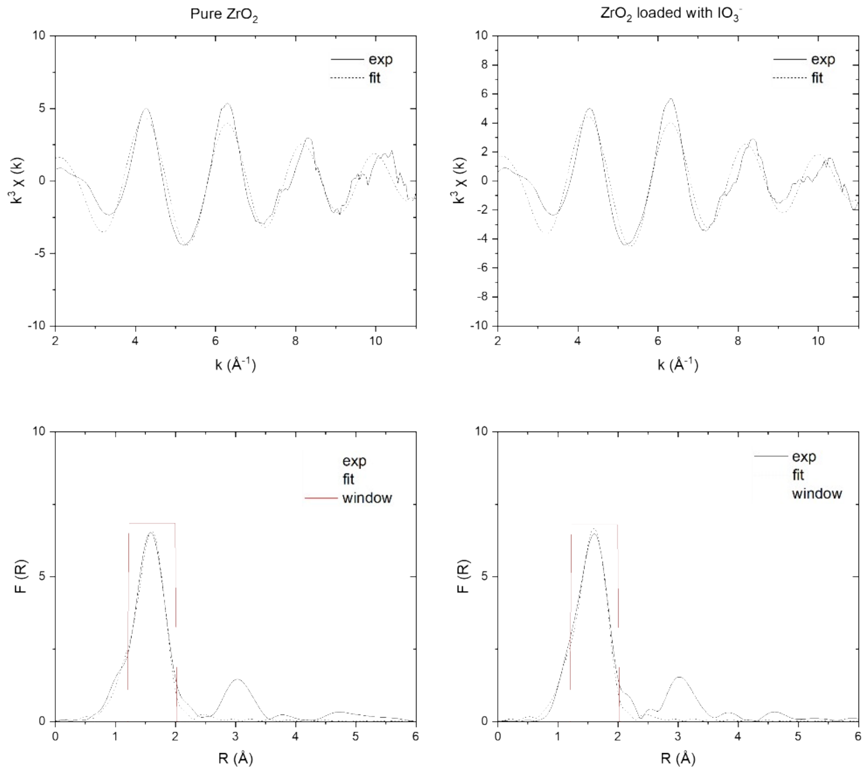

Figure 14 Zr K-edge EXAFS fits in R- and k-space for pure and loaded ZrO_2.

Table 3 Fitting parameters for Zr K-edge EXAFS spectra measured for pure and IO_3^- loaded ZrO_2 and $Zr(Sb)O_2$. S_0^2 (0.69 ± 0.03) and ΔE_0 were extracted from the fit of ZrO_2 sample and set as global parameters. The combined R-factor for the fit was 0.0163.

**Sample**	**ΔE_0 eV**	**R Å**	**N**	**σ^2**
**ZrO_2**				
Pure	-3.9(5)	2.15(1)	6 (fixed)	0.007(2)
IO_3^- loaded	-3.9(5)	2.15(1)	6.2(8)	0.008(1)
**$Zr(Sb)O_2$**				
Pure	-3.9(5)	2.15(1)	6.5(9)	0.007(1)
IO_3^- loaded	-3.9(5)	2.15(1)	6.2(8)	0.007(1)

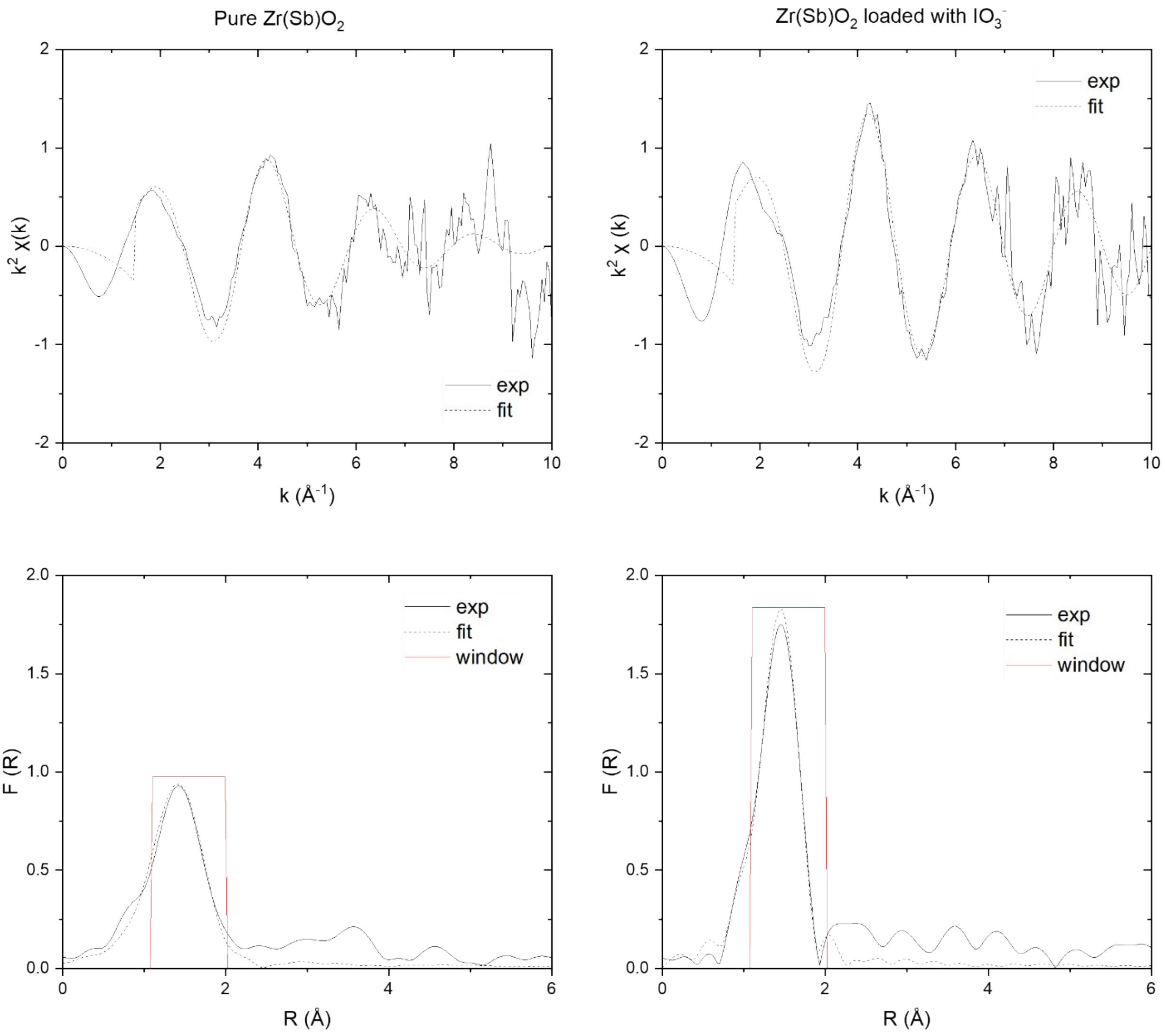

Antimony

Table 4 Fitting parameters for Sb K-edge EXAFS spectra measured for pure and IO_3^- loaded ZrO_2 and $Zr(Sb)O_2$. S_0^2 (0.74 ± 0.04) and ΔE_0 were extracted from the fit of Sb_2O_3 sample and set as global parameters. The combined R-factor for the fit was 0.012. R-space 1.1 – 2.0 Å based on k-space 2.7 – 8 $Å^{-1}$ (pure) or 2.7 – 10 $Å^{-1}$ (loaded).

Sample	ΔE eV	R Å	N	σ²
**$Zr(Sb)O_2$**				
Pure	8.1(6)	1.99(3)	6(2)	0.012(5)
IO_3^- loaded	8.1(6)	1.97(2)	6(1)	0.002(3)

Figure 15 Sb K-edge EXAFS fits in R- and k-space for pure and loaded Zr(Sb)O2.

3.4. GC-MS chromatograms

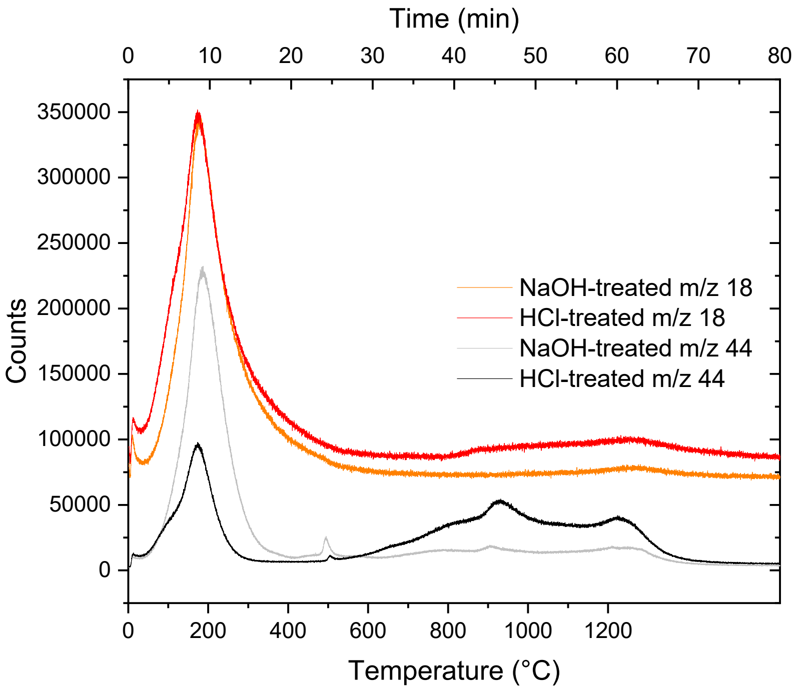

Figure 16 GC-MS chromatogram for m/z 18 (H_2O) and m/z 44 (CO_2) for HCl and NaOH treated $ZrSbO_2$.

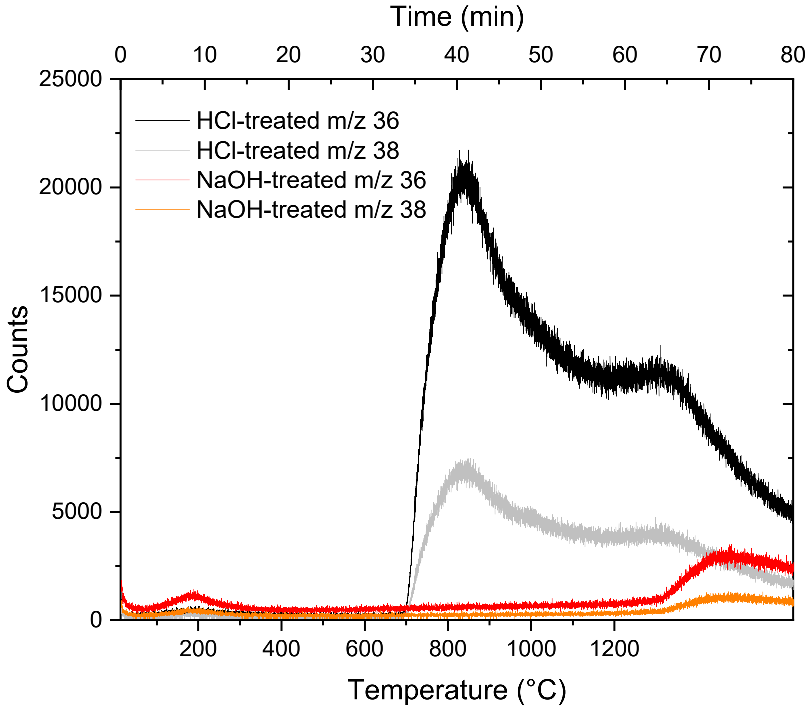

Figure 17 GC-MS chromatogram for m/z 36 ($H^{35}Cl$) and m/z 38 ($H^{37}Cl$) for HCl and NaOH treated $ZrSbO_2$.

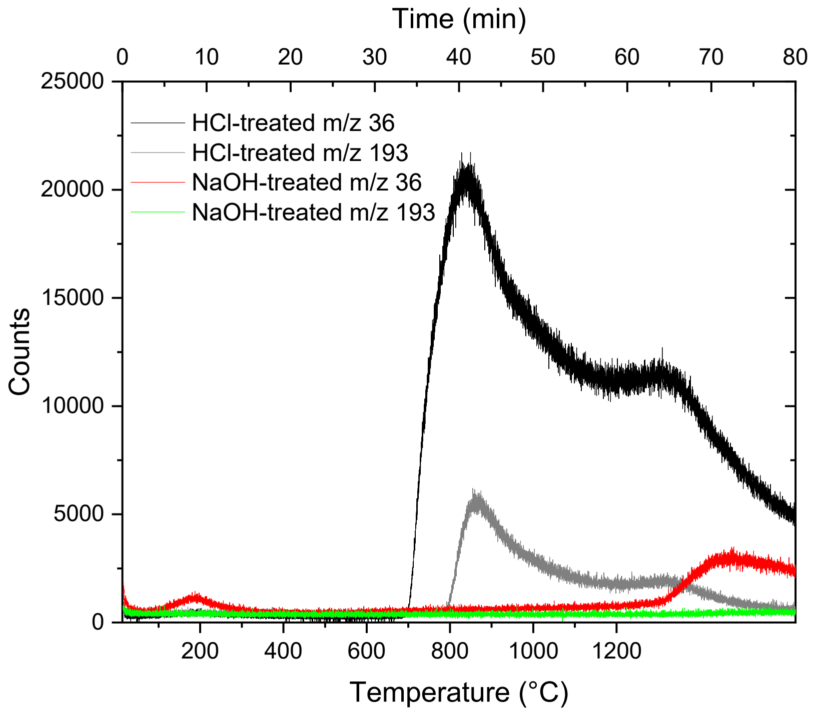

Figure 18 GC-MS chromatogram for m/z 36 (HCl) and m/z 193 ($SbCl_5$) for HCl and NaOH treated $ZrSbO_2$.

Acknowledgements

We thank Timo Hatanpää for his help with the TGA experiments and Bree Revill for her help with the regeneration column experiments. We acknowledge DESY (Hamburg, Germany), a member of the Helmholtz Association HGF, for the provision of experimental facilities. Parts of this research were carried out at PETRA III P64, and we would like to thank the beamline scientists Wolfgang Caliebe and Akhil Tayal for their efforts during the XAS measurements. Beamtime was allocated for proposal I-20200428 EC. SEM imaging and XRD measurements were done in ALD Center Finland research infrastructure. The financial support from Doctoral Program in Chemistry and Molecular Sciences (CHEMS) is gratefully acknowledged.
